# Assessing the interaction of oceanic and riverine processes on coastal phytoplankton dynamics in the East China Sea

**DOI:** 10.1007/s42995-024-00260-y

**Published:** 2025-01-07

**Authors:** Jiawei Gao, Rong Bi, Julian P. Sachs, Yaoyao Wang, Yang Ding, Hong Che, Jing Zhang, Peng Yao, Jie Shi, Meixun Zhao

**Affiliations:** 1 Frontiers Science Center for Deep Ocean Multispheres and Earth System, and Key Laboratory of Marine Chemistry Theory and Technology, Ministry of Education, Ocean University of China, Qingdao, 266100 China; 2 Laboratory for Marine Ecology and Environmental Science, Qingdao Marine Science and Technology Center, Qingdao, 266237 China; 3 School of Oceanography, University of Washington, Seattle, WA USA; 4 Laoshan Laboratory, Qingdao, 266237 China; 5 Faculty of Science, Academic Assembly, University of Toyama, Toyama, 9308555 Japan; 6 Key Laboratory of Marine Environment and Ecology, Ministry of Education, Ocean University of China, Qingdao, 266100 China

**Keywords:** Lipid biomarkers, Diatoms, Dinoflagellates, Changjiang diluted water, Kuroshio subsurface water, East China Sea

## Abstract

The interaction of riverine inputs and ocean current systems causes complex spatiotemporal variations in phytoplankton dynamics in marginal seas of the northwest Pacific Ocean, yet quantitative assessments of these variations and their causes remain limited. Here we evaluate phytoplankton biomass and community structure changes using lipid biomarkers, accompanying ocean circulation and nutrient variations in surface waters collected in spring and summer of 2017–2018 at 118 sites in the East China Sea off the Zhejiang coast. High biomass of diatoms, inferred from brassicasterol concentrations, shifted from the south in spring to the north in summer, while high dinoflagellate biomass, inferred from dinosterol concentrations, occurred mainly in the Changjiang (Yangtze) River plume and adjacent areas in both seasons. Seasonal variation in phytoplankton distribution was linked to the spatial extents of water masses such as the Changjiang Diluted Water (CDW) and the intrusion of the Kuroshio Subsurface Water (KSSW). A three end-member mixing model was applied to quantify water mass contributions. The results showed that an increase in the KSSW (from 0 to 40%) and a decrease in the CDW (from 100 to 20%) resulted in a significant (20%) increase in diatom proportions and a significant (20%) decrease in dinoflagellate proportions. Dinoflagellate proportions were highest in the CDW-dominated region, while diatoms and total phytoplankton biomass were higher in the CDW–KSSW mixing region and the KSSW-dominated region. This study highlights the dynamic response of the phytoplankton community to water mass changes in marginal seas that can aid coastal ecosystem management.

**Supplementary Information:**

The online version contains supplementary material available at 10.1007/s42995-024-00260-y.

## Introduction

**Fig. 1:**
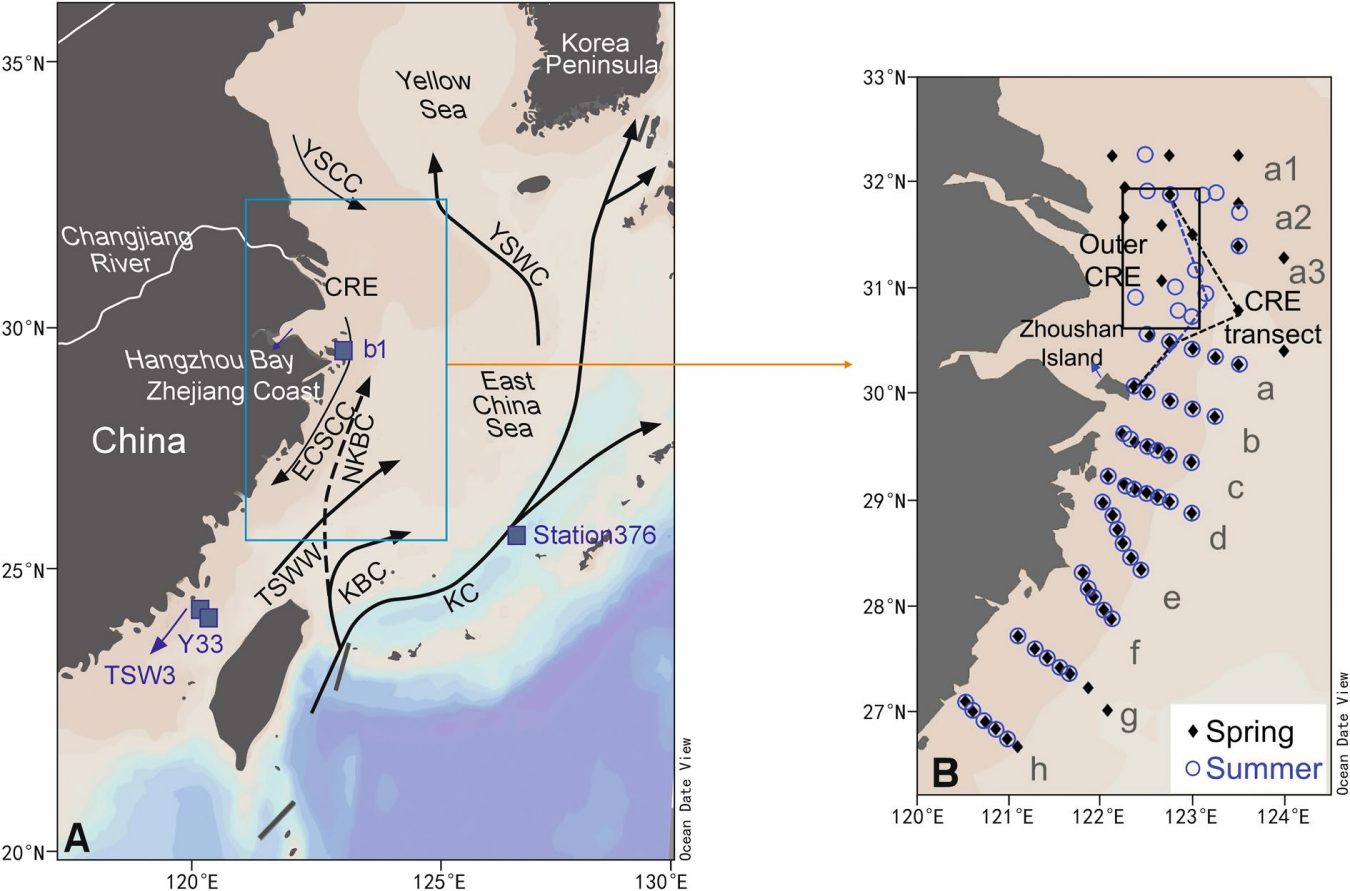
Maps of the study area with a simplified illustration of the shelf circulation system in summer (**A**) and sampling stations in spring 2017 and summer 2018 (**B**). KC: the Kuroshio Current; TC: the Tsushima Current; NKBC: the Nearshore Kuroshio Branch Current; KBC: the Kuroshio Branch Current; ECSCC: the East China Sea Coastal Current; YSCC: the Yellow Sea Coastal Current; TWC: the Taiwan Warm Current; TC: the Tsushima Current; CRE: the Changjiang River Estuary. The width of lines represents the strength of the currents. In panel **A**, the blue squares represent end-members of the Changjiang Diluted Water, the Kuroshio Subsurface Water, and the Taiwan Strait Water. In panel **B**, the letters a1–h denote sampling transects, with black and blue dashed lines representing the CRE transect in spring and summer, respectively. The black square delineates the region of outer CRE. This figure is modified from Bian et al. (2013) and Yang et al. (2021)

Marginal seas of the northwest Pacific Ocean (NWPO) are oceanographically and biologically dynamic owing to the interactions of the Kuroshio Current system, several large rivers, and the Asian Monsoon. In addition, they are subject to intense influences from anthropogenic activities and climate change (Liu et al. 2014; Song et al. 2020; Sun et al. 2022). Especially, in the East China Sea (ECS) coast of the Zhejiang province marine ecosystems are strongly affected by the interactions of river inputs and oceanic water masses (Chen 2008; Zhang et al. 2019, 2022; Zhou et al. 2018). The Changjiang (Yangtze) River accounts for more than 90% of terrestrial riverine inputs to the ECS and human activities have strongly impacted the timing and quantity of Changjiang discharge as well as its nutrient and particulate load, with the largest discharge occurring in summer, followed by spring and autumn (Dai et al. 2011; Jiang et al. 2014; Wang et al. 2021). The Nearshore Kuroshio Branch Current can reach the Zhejiang coast from spring to autumn and extend north to the Changjiang River Estuary (CRE), influencing the temperature, salinity, and nutrient concentrations of the ECS (Fig. 1). Invasion of Kuroshio Subsurface water (KSSW), which is mainly regulated by natural climatic forcing such as the Pacific Decadal Oscillation (PDO), also affects nutrient concentrations in the ECS (Andres et al. 2009; Fedele et al. 2021). The KSSW can upwell to the surface along the Zhejiang Coast as early as April (Yang et al. 2018) and it can intrude into the 50-m isobath where it comprises 20 to 40% of the water in spring (Che et al. 2022). The Changjiang Diluted Water (CDW) and upwelling induced by the KSSW have been shown to be important factors regulating phytoplankton growth in the Zhejiang coastal area (Xu et al. 2020; Zhang et al. 2019).

The direct effect of the CDW on phytoplankton has been widely studied in the western ECS. High turbidity induced by the CDW generally limits the growth of phytoplankton in the CRE (Tseng et al. 2014; Wang et al. 2019a, b), while high phytoplankton productivity tends to occur outside of the turbidity front in the outer estuary (Chai et al. 2006; Wang et al. 2017). Phytoplankton community composition shows a clear seasonal succession in the Changjiang River plume, which is related to environmental variability and biological interactions. For example, in early spring when nutrient concentrations are high and temperatures begin to rise, the Changjiang River plume is often dominated by diatoms (Yu et al. 2017). In late spring, nutrients, especially dissolved inorganic phosphorus (DIP) that is supplied primarily by the CDW, become limiting for phytoplankton growth. Thus, the high N/P of CDW, rising temperatures, and the dissolved organic matter produced after the spring bloom of diatoms may be conducive to dinoflagellate growth (Li et al. 2009; Yu et al. 2017). Overall, dinoflagellate biomass or abundance in spring is higher than that of diatoms in the Changjiang River plume (Guo et al. 2014; Liu et al. 2016; Yue et al. 2021; Zhao et al. 2015), while in other seasons diatom biomass is higher than that of dinoflagellates (Liu et al. 2016). Thus, high nutrient concentrations and turbidity associated with the CDW cause complex spatiotemporal variations in phytoplankton productivity and community structure in regions impacted by this water mass.

In addition to the CDW influence on phytoplankton, upwelling mainly induced by winds, tide, topography, and the KSSW brings higher concentrations of DIP to the Zhejiang coast and the CRE (Che et al. 2022; Hu and Wang 2016; Xu et al. 2018, 2020; Yang et al. 2013). Outcrops of the KSSW cause high phytoplankton productivity outside the CRE (Tseng et al. 2014) and regulate phytoplankton community structure. Algal blooms in 2005 were attributed to the KSSW and its associated delivery of DIP, favored dinoflagellate growth (Zhou et al. 2019). In contrast, other studies based on pigment measurements indicated that diatoms were more dominant during high-DIP years on the mid-shelf of the ECS, suggesting upwelling of KSSW as the main cause of diatoms flourishing (Xu et al. 2019). More recently, a coupled physical–biological modeling study showed that the CDW- and KSSW-derived nutrients governed algal blooms north and south of the Zhoushan Islands, respectively, in the CRE and adjacent areas (Xu et al. 2020). Despite these efforts in studying the effects of KSSW intrusion, the interplay between the CDW and the KSSW on phytoplankton biomass and community structure remains unclear and poorly quantified, mainly due to the lack of high-resolution observations in the Zhejiang coastal waters.

Lipid biomarkers are source specific and have high chemical stability, making them useful proxies of phytoplankton biomass and community structure in both paleoenvironments and the contemporary ocean (Schubert et al. 1998; Wang et al. 2022; Wu et al. 2016; Zhao et al. 2006). Previous studies on lipid biomarkers in suspended particles have provided insights into the control of water masses on spatiotemporal variations of phytoplankton in the continental shelf and open ocean, including the Yellow Sea (Cao et al. 2022; Wu et al. 2016), the ECS (Bi et al. 2018; Wu et al. 2016), the South China Sea (Dong et al. 2015), and the NWPO (Wang et al. 2022). To date, there are insufficient high-resolution observations of lipid biomarker composition and distribution in suspended particles from coastal upwelling systems, which are important sites to evaluate the effects of human-induced and natural climate forcing on phytoplankton ecology.

Here, we use lipid biomarkers from high-resolution observations (the average horizontal distance between adjacent profiles and adjacent station was about 15 km) to quantitatively assess the interplay between the CDW and the KSSW on phytoplankton biomass and community structure in the spring (May 4–17 of 2017) and summer (August 5–19 of 2018), bloom months in the Zhejiang coastal waters of the ECS (Table S1). The late spring (May) and late summer (August) were chosen as the study periods, since the CDW increases from spring to summer and the KSSW upwells to the surface water in spring and summer. Also, harmful algal blooms often occur between spring and autumn in the ECS (Li et al. 2014a, b; Liu et al. 2013). Diatoms and dinoflagellates are the predominant phytoplankton groups in the ECS, with haptophytes comprising a minor component (Guo et al. 2020; Jiang et al. 2015; Shih et al. 2019). According to previous studies of dominant algal species and their sterol composition in the ECS (Table S2), brassicasterol, dinosterol, and C_37_ alkenones can be used as biomass proxies for diatoms, dinoflagellates, and haptophytes, respectively. The sum of brassicasterol, dinosterol, and C_37_ alkenones (∑PB) can be applied as a proxy of total phytoplankton biomass, with the ratio of each biomarker to ∑PB denoting the contribution of each phytoplankton group to total phytoplankton biomass. We hypothesize that (1) the CDW favors the predominance of dinoflagellates mostly within the CRE and the KSSW favors the predominance of diatoms mostly within the upwelling areas, and (2) the interaction of the CDW and the KSSW controls total phytoplankton biomass and the co-occurrence of diatom and dinoflagellate blooms during times and in regions when the two water masses interact.

## Materials and methods

### Study area and sampling

The study area is located in the Zhejiang coastal waters of the western ECS where the current system is complex (Fig. 1A) (Bian et al. 2013; Yang et al. 2021). This area is influenced by several water masses, including the CDW, the KSSW, the Taiwan Strait Water (TSW), and the mixture of the three water masses, i.e., the shelf mixed water, with the CDW and the KSSW being major contributors of nutrients (Chen 1996; Zhang et al. 2019). The low-salinity and high-nutrient CDW extends from the CRE to the ECS outer shelf. The high-salinity and high-DIP KSSW upwells in April and weakens in October along the Zhejiang coast (Wang et al. 2018; Yang et al. 2018). In warm seasons, the Taiwan Warm Current originates from the TSW and the Kuroshio Current intrusion water, with the former derived from the warm and low-nutrient South China Sea Warm Current Water and the latter derived from the cold and high-nutrient KSSW (Bian et al. 2013).

Two cruises were conducted in spring of 2017 (NORC2017–03 from May 11 to May 17, 2017 and MZ17SP from May 4 to May 11, 2017) and one cruise was conducted in summer of 2018 (MZ18SU from August 5 to August 19, 2018) (Table S1). A total of 118 stations with water depths of less than 100 m were sampled between the latitudes of 26.6–32.5°N and longitudes of 120–124°E (Fig. 1B).

Suspended particle samples for lipid biomarker analyses and TSM (total suspended matter) were collected from surface seawater by Whatman GF/F filters (diameter: 150 mm; water volume: 10–50 L) for 61 and 57 stations in May and August, respectively (Table S1). Water samples for nutrient measurements were collected through cellulose acetate membranes at 37 and 57 stations in May and August, respectively, at the depths similar to those of suspended particle samples (Table S1).

### Sample analysis

Temperature, salinity, and chlorophyll *a* (Chl *a*) were measured in situ using CTD instruments (SBE 25, Sea Bird Electronics Inc., USA) during cruise NORC2017–03, RBR concerto logger (RBR Ltd., Ottawa, Canada) during MZ17SP and RBR XR–620 (RBR Ltd., Ottawa, Canada) during MZ18SU, respectively. Prior to the cruises, all sensors were calibrated and CTD-based Chl *a* fluorescence was converted to Chl *a* concentrations in μg L^–1^, and Chl *a* data for the summer cruise was previously used to study the regulation of coastal upwelling and river plume on hypoxia in our study region (Wei et al. 2021). In addition to the CTD-based fluorescence Chl *a*, Chl *a* for 39 stations in spring was also measured using a fluorophotometer following extraction with 90% acetone. A significant correlation between the solvent-extracted and CTD-based Chl *a* concentrations was observed (*n* = 39, R^2^ = 0.555, *p* < 0.001), indicating nonsignificant difference between the two methods. Thus, the CTD-based Chl *a* concentration data for both seasons are presented in the results.

The concentrations of NO_x_ (NO_2_^–^ + NO_3_^–^) and DIP were measured with a 4802 UV/VIS Double Beam Spectrophotometer (UNIC, China) and a SAN Plus continuous-flow analyzer (Skalar Analytical B.V., the Netherlands), respectively, for samples from the cruise NORC2017–03. An AA3 continuous-flow analyzer (SEAL Analytical GmbH, Germany) was used for nutrient analyses for cruise MZ17SP, and a QUAATRO continuous-flow analyzer (SEAL Analytical GmbH, Germany) was used for nutrient analyses for cruise MZ18SU. According to Ward et al. (1990), the concentration of DIP was set to 0.01 μmol L^–1^ when it was lower than the detection limits (cruise: MZ17SP; 0.02 μmol L^–1^) in May. It should be noted that the concentration of silicate (0.51–69.94 μmol L^–1^) was also measured and not shown in this study, as silicate concentration is usually high and not limited in our study area (Wang et al. 2003).

TSM concentrations were first determined gravimetrically and subsequently lipid biomarkers were analyzed for each filter. Brassicasterol, dinosterol, and C_37_ alkenones were measured according to Zhao et al. (2000), with C_19_ *n*-alkanol being added as an internal standard. Organic matter in the freeze-dried samples was extracted using the mixture of dichloromethane and MeOH (3:1, v/v), hydrolyzed with 6% KOH in MeOH, and separated into polar and nonpolar components using silica gel column chromatography. After derivatization using *N,O*-bis(trimethylsily)-trifluoroacetamide (BSTFA, at 70 °C for 1 h), sterols, and C_37_ alkenones were measured by gas chromatography (Agilent 7890 N) with a flame ionization detector (FID) and an HP-1 capillary column (50 m × 0.32 mm × 0.17 μm). Lipid biomarkers were quantified by comparing peak areas to that of the internal standard. To control the quality of lipid biomarker analysis, we repeated to measure one lab standard per 20–30 measurements of lipid biomarkers on gas chromatography through the course of our study. There were totally 15 repeated measurements. Because of the low concentrations of lipid biomarkers in our study region, it was not possible to have replicates at each station. The standard deviation (SD) of repeated measurements (*n* = 15) for brassicasterol/∑PB (B/∑PB), dinosterol/∑PB (D/∑PB), and C_37_ alkenones/∑PB (A/∑PB) was 1%, 2%, and 2%, respectively.

### Data analysis

**Fig. 2:**
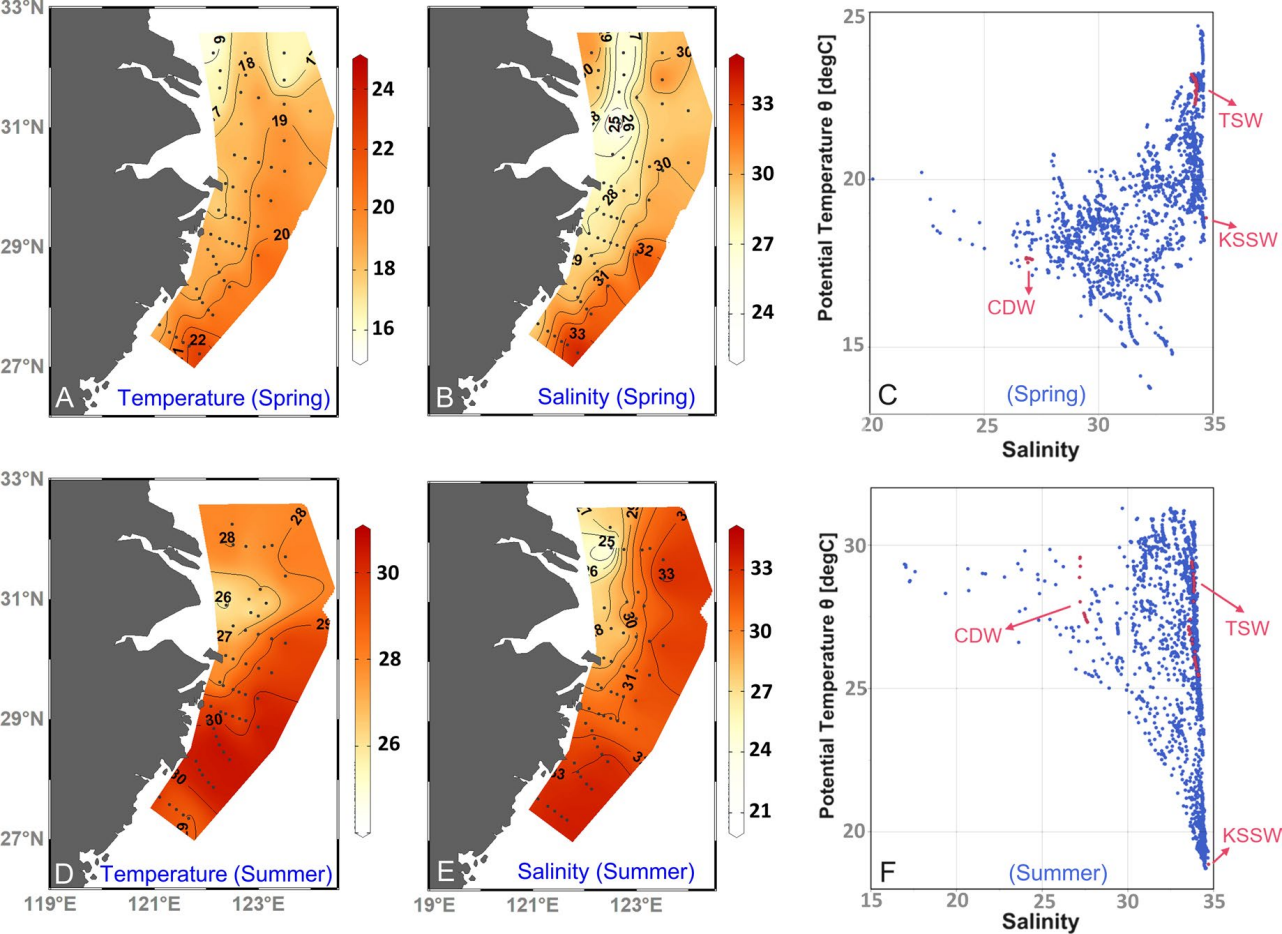
Distribution of temperature (**C**, **A** and **D**) and salinity (**B** and **E**) at 5 m, and the temperature–salinity diagram for all depths (1-m interval) in spring 2017 (**C**) and summer 2018 (**F**). The red points in panels **C** and **F** are the end-members of the CDW (Changjiang Diluted Water), the TSW (Taiwan Strait Water), and the KSSW (Kuroshio Subsurface Water)

A three end-member mixing model was applied to identify the areas influenced by the dominant water masses (the CDW and the KSSW) and to quantify the effects of the two water masses on phytoplankton based on conservation of both heat (temperature) and mass (salinity) (Han et al. 2012; Wang et al. 2016b), where the MATLAB package (Bosch et al. 2015) was adapted using a Bayesian Markov chain Monte Carlo method. Similarly, three end-member models based on mass balance of temperature and salinity have been also used in previous studies (Cao et al. 2011; Han et al. 2012; Wang et al. 2016b), and they can successfully reveal the biogeochemical processes in highly dynamic coastal ecosystems under the influence of river inputs and upwelling processes. Thus, this model can be used to assess the interaction of oceanic and riverine processes on coastal phytoplankton dynamics in our study. The end-member values for the CDW, the KSSW, and the TSW were selected based on T–S properties and the sample locations (Fig. 2; Table 1; Table S3) (Qi et al. 2014; Wang et al. 2016b; Zhou et al. 2018). According to the T–S diagram, three water masses were identified, i.e., the CDW, the TSW, and the KSSW (Fig. 2C, F). The CDW is characterized by low salinity (< 30), with low temperature in spring and high temperature in summer (Qi et al. 2014; Zhou et al. 2018). The TSW has high salinity (around 34.1) and high temperature both in spring and summer (Che and Zhang 2018; Qi et al. 2017). The KSSW, with a temperature of ~ 19 °C and salinity of 34.7, originates from a depth of 130–300 m along 24.9°N northeast of Taiwan (Qi et al. 2017; Yang et al. 2013). The actual end-member properties of the water masses that were used to calculate the percentage contribution of each water mass were set according to previous studies on end-member properties of the three water masses (Table 1 and Table S3 for the surface and bottom layer, respectively) (Che et al. 2022; Liu et al. 2022; Zhou et al. 2018). Proportions of water masses in the surface and bottom layer were calculated based on the water properties at the depth of 5 ± 1 m and 2 ± 1 m above the bottom, respectively, and stations out of the ranges of end-members were excluded. The average SD for the calculated proportions of CDW, TSWW, and KSSW at all stations was each 1% in spring and 2% in summer. It should be noted that the calculation of water mass contributions in our study did not consider diapycnal and isopycnal mixing processes. Furthermore, SST in summer is less conservative due to direct surface heating, causing uncertainties in the calculation of the proportions of TSW and KSSW. For example, KSSW proportions in summer may be underestimated due to the strong surface heating.

**Table 1:** Summary of the end-member properties used in the three end-member mixing model to calculate water mass proportions at the surface layer

	Spring			Summer		
Water mass	CDW ^b^	TSW ^c^	KSSW ^d^	CDW ^b^	TSW ^e^	KSSW ^d^
Representative Station	b1	TWS3	376	b1	Y33	376
Layer	Surface	Surface	Subsurface	Surface	Surface	Subsurface
Longitude (°E)	122.36	119.67	126.33	122.36	119.80	126.33
Latitude (°N)	30.07	25.00	26.74	30.07	24.84	26.74
Depth (m)^a^	5 ± 1	5 ± 1	200	5 ± 1	5 ± 1	200
Salinity^a^	26.85 ± 0.05	34.12 ± 0.11	34.7 ± 0.1	27.28 ± 0.14	33.73 ± 0.02	34.7 ± 0.1
Temperature (℃)^a^	17.67 ± 0.01	23.05 ± 0.14	18.9 ± 0.1	28.26 ± 0.66	29.39 ± 0.05	18.9 ± 0.1
NO_x_ (μmol L^–1^)	32.43	0.03	3.69	27.10	0.23	3.69
DIP (μmol L^–1^)	0.23	0.01	0.55	0.89	0.01	0.55

*CDW* changjiang diluted water, *TSW* Taiwan strait water, *KSSW* Kuroshio subsurface water

^a^For depth, salinity, and temperature, mean values (at 4–6 m depth for salinity and temperature) ± SD are shown

^b^The end-member values for CDW were obtained from the present study

^c^The end-member values for TSW in spring were obtained from cruise MZ17SP

^d^The end-member values for KSSW were obtained from Wang et al. (2016b)

^e^The end-member values for TSW in summer were obtained from cruise NORC2018-04

**Fig. 3:**
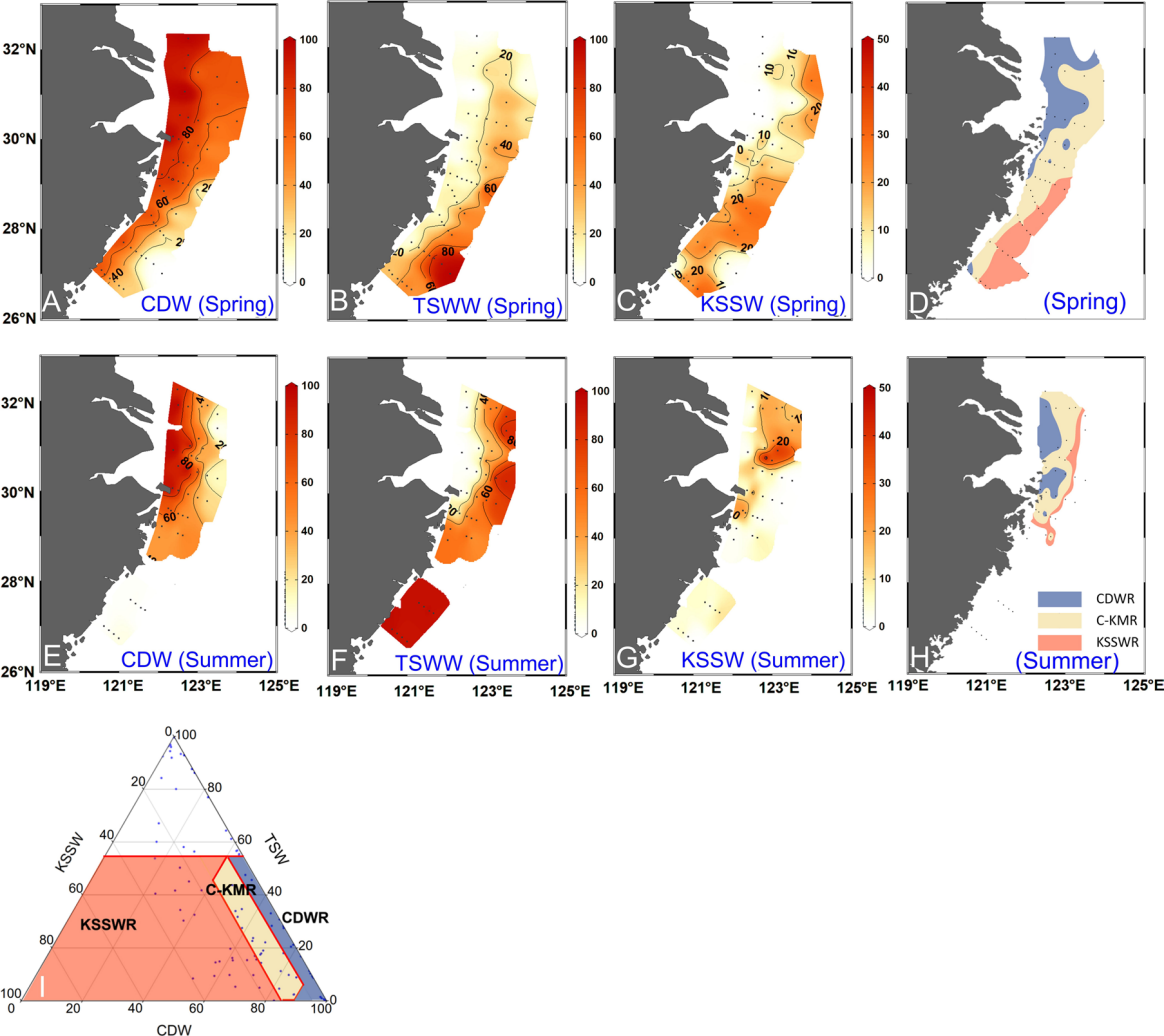
Proportions of three water masses in the surface water (**A**–**C**, **E**–**G**) and subregions identified based on water mass proportions (%) and their spatial distributions in spring 2017 and summer 2018 (**D**, **H**, **I**). In panel I, the white triangular area represents the TSW-dominant region (TSWR), with the remainder dominated by the KSSW and the CDW. The dark blue area in panels **D** and **H** represents the CDW-dominated region (CDWR), and the yellow and orange areas represent the CDW–KSSW mixing region (C-KMR) and the KSSW-dominated region (KSSWR), respectively. In panels **D** and **H**, sites within the TSWR and outside the range of end-members were excluded. For water mass abbreviations refer to the caption of Fig. 2

**Fig. 4:**
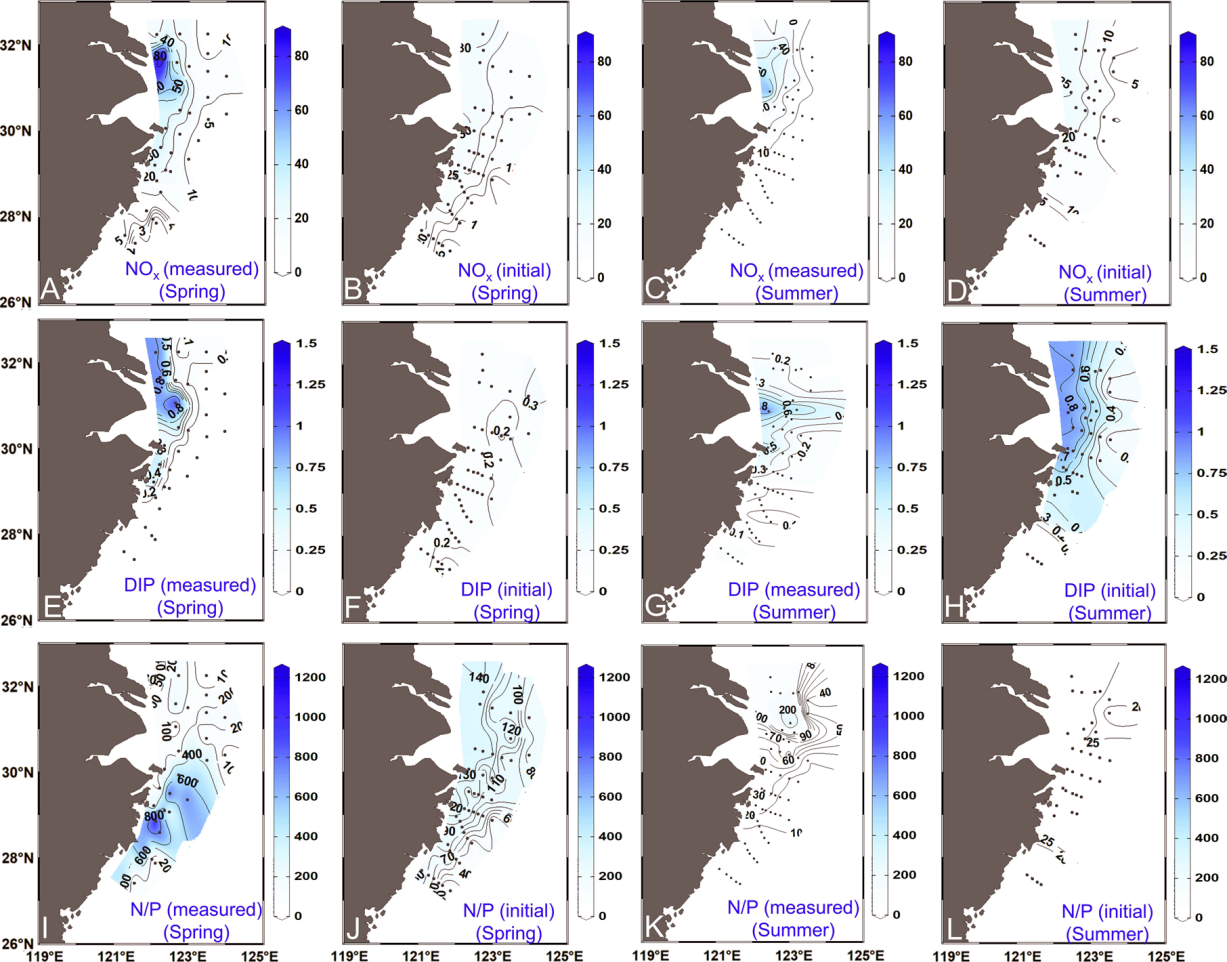
Distributions of NO_x_ (NO_2_^–^ + NO_3_^–^, μmol L^–1^), DIP (phosphate; μmol L^–1^), and N/P molar ratios in the surface water in spring 2017 and summer 2018. Measured values are shown in panels **A**, **E**, **I**, **C**, **G**, and **K**. Initial (i.e., preformed) values calculated based on the three end-member model results are shown in panels **B**, **F**, **J**, **D**, **H**, and **L**. The difference between the calculated and measured values is greatest in regions with high phytoplankton biomass

**Fig. 5:**
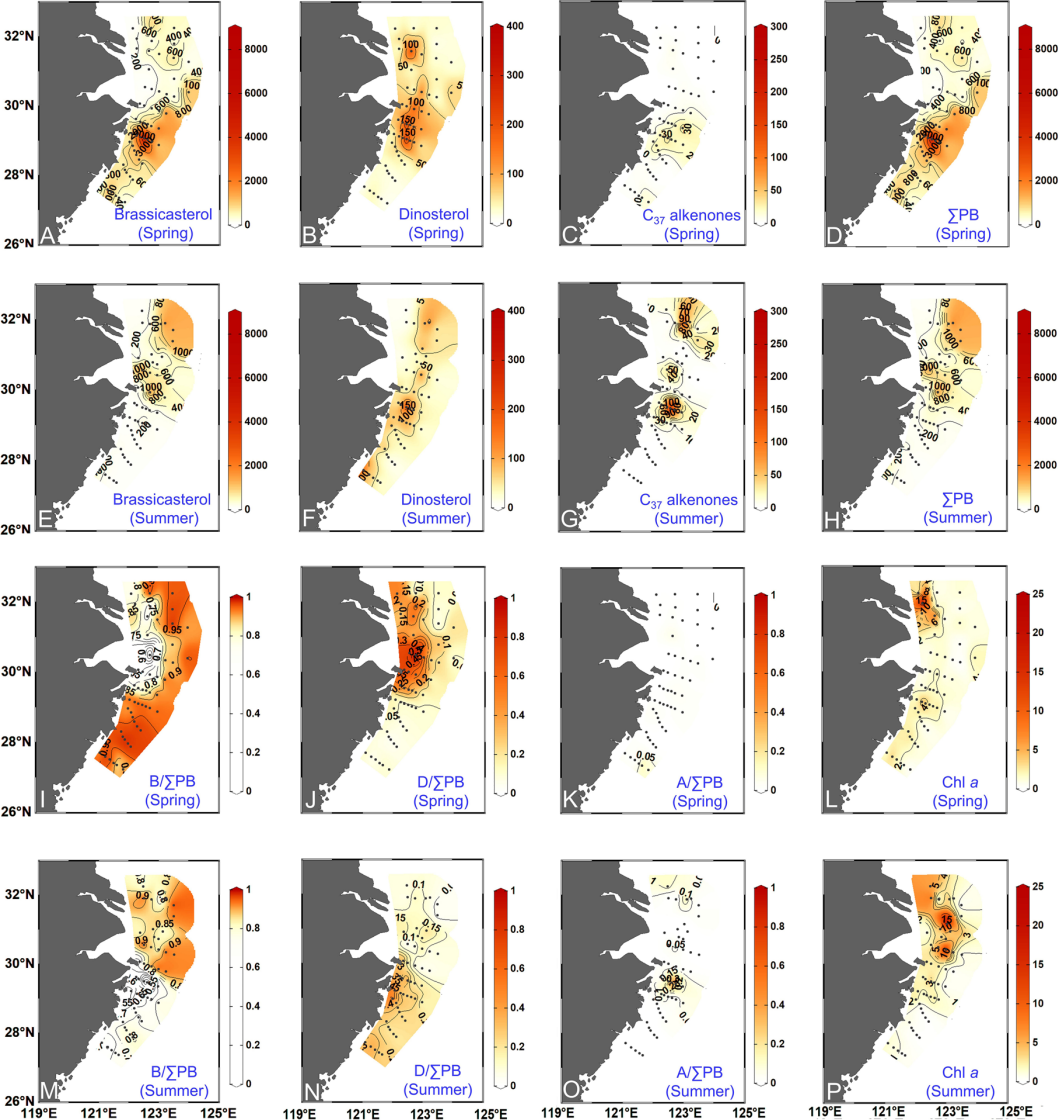
Maps of phytoplankton biomass and community structure. Distributions of brassicasterol (ng L^–1^, **A** and **E**), dinosterol (ng L^–1^, **B** and **F**), C_37_ alkenones (ng L^–1^, **C** and **G**), total lipid biomarkers (ng L^–1^, **D** and **H**) (∑PB: brassicasterol + dinosterol + C_37_ alkenones), B/∑PB (brassicasterol/∑PB, **I** and **M**), D/∑PB (dinosterol/∑PB, **J** and **N**), A/∑PB (C_37_ alkenones /∑PB, **K** and **O**), and chlorophyll* a* (μg L^–1^, **L** and **P**) in surface suspended particles in spring 2017 and summer 2018

The proportions of each water mass present at each sample location were then used to predict the initial nutrient concentrations (NO_x_ and DIP) in the surface and bottom waters that would result from conservative mixing of the water masses based on nutrient concentrations in the three end-members (Table 1; Figs. 3, 4, S1 & S2) (Che et al. 2022; Che and Zhang 2018; Zhou et al. 2018). Overall, the calculated nutrient concentrations had similar distribution patterns as the measured ones, showing high NO_x_ and DIP in the outer CRE and Hangzhou Bay (Fig. 4). However, the distribution of initial N/P molar ratios differed from measured values in spring, with calculated initial N/P being high in the outer CRE, outside Hangzhou Bay and adjacent areas in spring, and lower in summer (Fig. 4J, L). The initial nutrient concentrations reflect the original values supplied by water masses, while the measured values represent those after uptake of nutrients by phytoplankton and organic matter mineralization, so the two are not expected to agree, especially where algal productivity is high. Specifically, a higher measured nutrient concentration compared to that predicted from water mass mixing (i.e., physical processes) alone implies nutrient production process by organic matter mineralization. In contrast, a higher predicted nutrient concentration based on water mass mixing compared to the measured value implies nutrient consumption by phytoplankton. The latter is the case, for example, in the southern region off the Zhejiang coast in spring when phytoplankton biomass is high (Figs. 4C; 5L) (Guo et al. 2014).

Notwithstanding some expected differences in the calculated preformed (initial) and measured nutrient concentrations owing to nutrient utilization by phytoplankton and organic matter mineralization, we compared the former with the latter, reasoning that, at least in locations with low-to-moderate phytoplankton productivity and/or very high preformed nutrient concentrations, the two values should be similar (Figs. 4 & S2). Significant positive correlations exist between the initial and measured concentrations of NO_x_ (and DIP). Spearman’s correlation analysis was conducted to quantitatively assess the relationship between nutrient concentrations (in situ measured and model-derived values) and Chl *a* concentrations in both seasons using IBM SPSS 25 software. Significant positive correlations exist between initial nutrient concentrations and Chl *a* concentration in both seasons (Table S4). This provides some confidence that the water mass mixing model based on T–S properties of CDW, KSSW, and TSW can be used to infer the fractional contribution of each water mass at the sampling locations, and by extension the preformed nutrient concentrations at those locations.

**Fig. 6:**
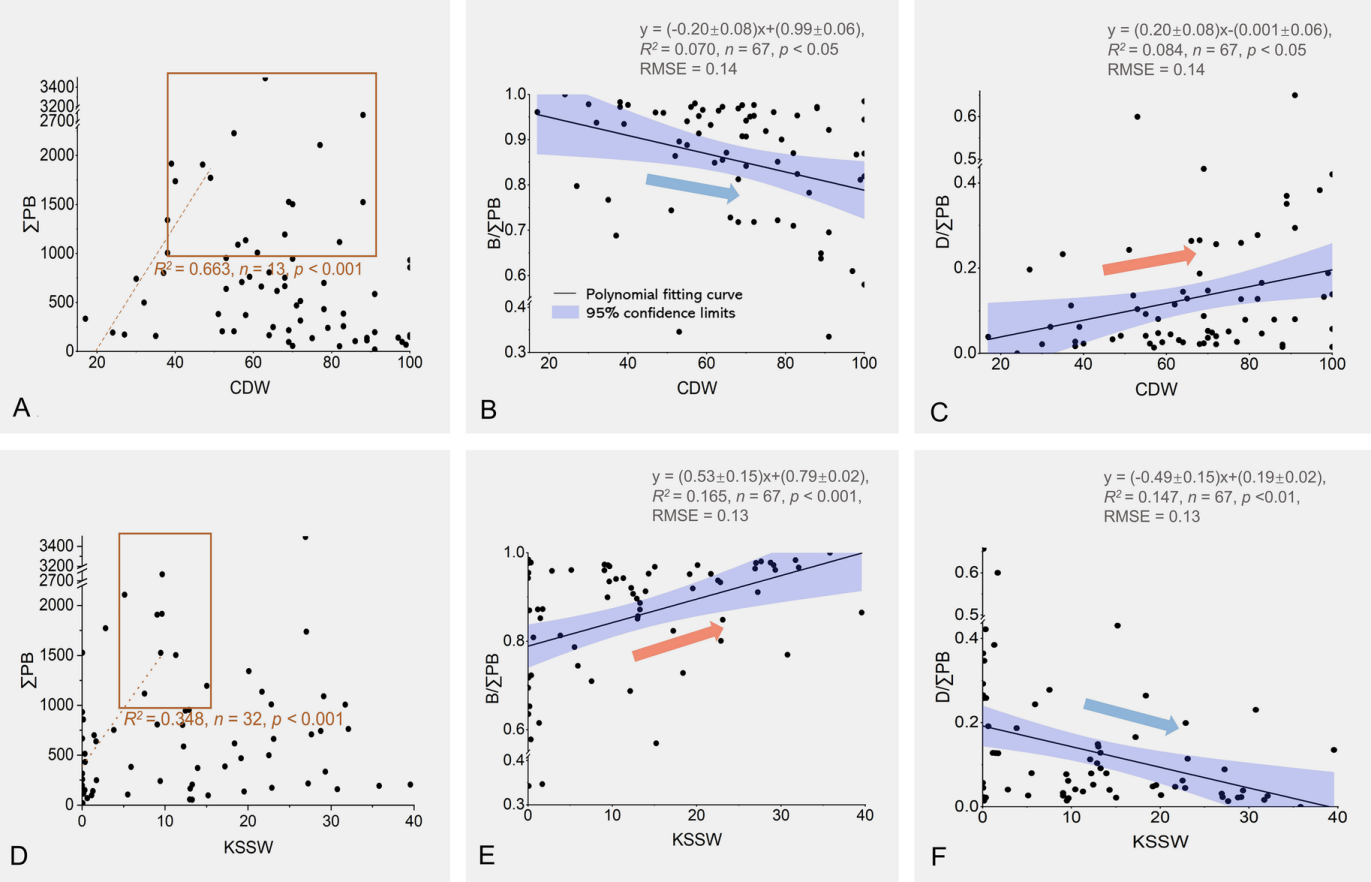
Lipid biomarker concentrations (ng L^–1^) and their proportions in response to the changes in the proportions (%) of CDW (**A**–**C**) and KSSW (**D**–**F**). In panels **A** and **D**, the overall correlations between ∑PB and water mass proportions are not significant, while there are significant positive correlations when water mass proportions are < 50% for the CDW and < 10% for the KSSW, as indicated by the red dotted lines. Red boxes in panels **A** and **D** are added to visually highlight high ∑PB in the mixing zone of CDW and KSSW. In panels **B**, **C**, **E**, and **F**, the blue shaded areas represent the 95% confidence intervals, with the orange and blue arrows indicating the significant increase and decrease in lipid biomarker proportions, respectively. Sampling stations in the TSWR subregion were excluded, as was station d4 with extremely high ∑PB (8589 ng L^–1^). RMSE: root mean square error. For water mass and lipid biomarker abbreviations refer to the captions of Fig. 2 and Fig. 5, respectivley

**Fig. 7:**
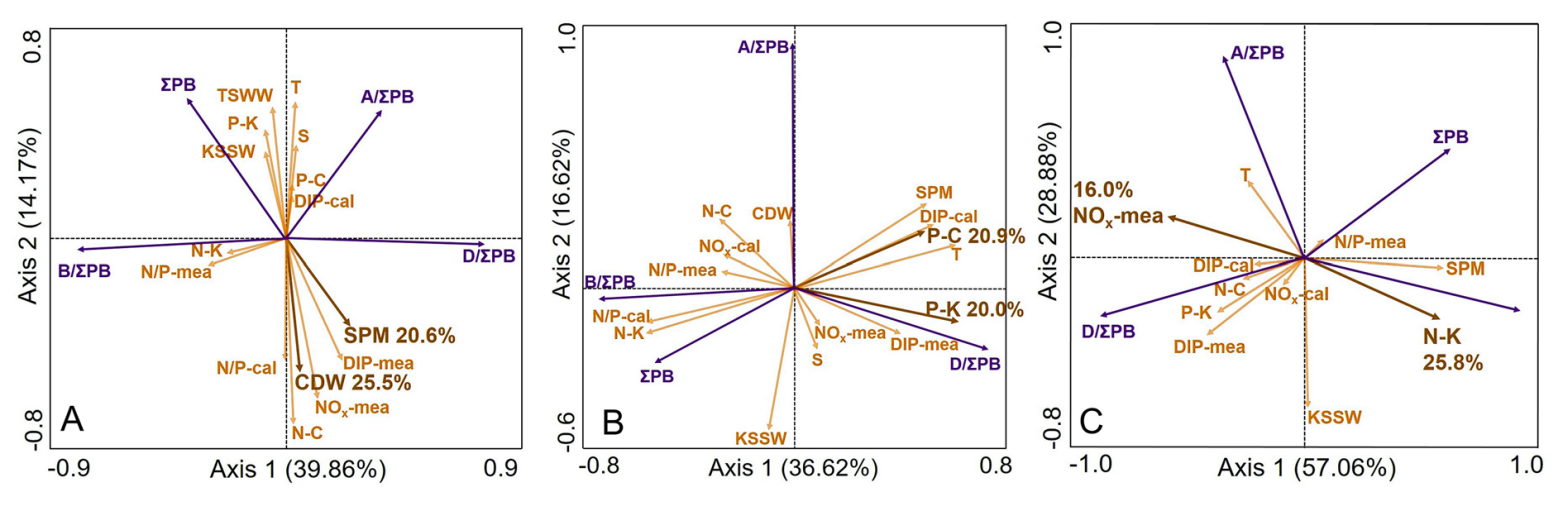
Redundancy Analysis (RDA) of hydrographic parameters, nutrients, and lipid biomarkers in the three subregions, i.e., the CDWR (**A**), the C-KMR (**B**), and the KSSWR (**C**). Blue and red arrows represent lipid biomarkers and hydrographic parameters, respectively, with dark red arrows showing the two parameters contributing the most and their contributing proportions in the RDA also shown. DIP-mea, NO_x_-mea, and N/P-mea: DIP, NO_x_, and NO_x_/DIP measured during the cruises; DIP-cal, NO_x_-cal, and N/P-cal: DIP, NO_x_, and NO_x_/DIP calculated from water mass composition; C-P (or C-N) and K-P (or K-N): the calculated proportion of DIP (or NO_x_) provided by the CDW and the KSSW, respectively. For water mass, subregion, and lipid biomarker abbreviations refer to the captions of Figs. 2, 3, and 5, respectivley

To analyze the effects of dominant water masses (the CDW and the KSSW) in our study area, the TSW-dominated stations (TSW proportion ≥ 55%) were excluded and the data from both seasons were combined. It should be noted that the TSW has much less contributions to nutrient composition compared to the CDW and the KSSW in our study region (Chen 1996; Zhang et al. 2019); thus, the effects of major water masses, the CDW and KSSW, are mainly discussed in this study. Linear or polynomial curves were fitted to the biomarker data to evaluate the relationship, if any, between biomarkers and water masses (Figs. 6, 7) (B/∑PB: brassicasterol/∑PB; D/∑PB: dinosterol/∑PB; A/∑PB: C_37_ alkenones/∑PB) using Origin software (version: Origin 2018). Linear regressions were selected when the *p* value and the root mean square error (RMSE) were lower than polynomial regressions since we had no a priori reason to believe one model is better than the other (Cui and Schubert 2016). Based on the results of regressions, water mass proportions, and their geographical distributions, three subregions were identified in spring and summer, respectively, i.e., the CDW-dominated region (CDWR; 22 sites; CDW > 40%, KSSW ≤ 10%), the CDW–KSSW mixing region (C-KMR, 20 sites; 40% ≤ CDW ≤ 90%, 5% ≤ KSSW ≤ 15%), and the KSSW-dominated region (KSSWR; 26 sites; CDW < 90%, KSSW > 5%). The Kruskal–Wallis rank sum test was applied to test the similarity of ∑PB, B/∑PB, D/∑PB, and A/∑PB between the three subregions in order to determine whether the identification of the three subregions was reasonable. Prior to the similarity test, data were tested for normality and homogeneity of variances via a Shapiro–Wilk test and a Levene's test, respectively, in the three subregions. As the data of all variables tested were not normally distributed (Table S5) and variances were not homogeneous for most of the variables (Table S6), the Kruskal–Wallis rank sum test was chosen, and the results showed significant differences between the three subregions (Table S6). The Kruskal–Wallis rank sum test, Shapiro–Wilk test, and Levene's test were performed using IBM SPSS 25 software.

The effects of hydrological parameters (and nutrients) of different water masses on lipid biomarker concentrations (and their ratios) in each subregion were determined via Redundancy Analysis (RDA) using Canoco software (version: Canoco 5). Data from both seasons were combined in each subregion, as water masses control phytoplankton biomass and community structure in both seasons.

The significance level in all cases was *p* < 0.05.

## Results

### Distributions of temperature, salinity, water masses, and nutrient composition

At surface layer, temperature was 19.1 ± 1.6℃ (mean ± SD) in spring and 28.9 ± 1.5℃ in summer (Fig. 2A, D; Table S1). Salinity was 29.7 ± 2.1 in spring and 31.3 ± 2.4 in summer (Fig. 2B, E). In spring, the cold, fresh CDW (average proportion: 59%) spread southward from the CRE across a large area along the coast, while the warm, high-salinity TSW (average proportion: 27%) occurred in the southeastern part of the study area (Figs. 2A, B; Fig. 3A, B). High proportions of the KSSW (average proportion: 13%) occurred in the southeast of the study area. In summer, the TSW (average proportion: 50%) with high temperature and salinity was observed in the south (Fig. 2D, E), and the CDW (average proportion: 43%) covered a smaller area than in spring (Fig. 3A, E). High proportions of the KSSW (average proportion: 7%) occurred at several sites in the vicinity of the CRE (Fig. S1F).

To infer the influence of upwelling, the distribution of temperature and salinity was analyzed along two transects (the CRE transect and the f transect) where the KSSW usually upwells to the surface (Fig. 1B) (Yang et al. 2013, 2018). Along the CRE transect, cold and fresh water occurred at the surface and high-salinity water occurred at the bottom layer in spring (Figs.1B; S3B, C), while the apparent outcrop of cold and high-salinity water was found at the surface in summer (Fig. S3F, G). Along the f transect, temperature and salinity increased seaward in spring (Fig. S3D, E), and both were high at the surface layer at most sites in summer (Fig. S3H, I).

NO_x_ at the surface layer was 17.79 ± 17.93 μmol L^–1^ in spring and 8.20 ± 14.83 μmol L^–1^ in summer (Fig. 4A, C; Table S1). DIP was 0.16 ± 0.28 μmol L^–1^ in spring and from 0.05 to 1.37 μmol L^–1^ (average: 0.22 μmol L^–1^) in summer (Fig. 4E, G). N/P molar ratios had a larger range in spring (5–1283) than that in summer (0.7–658) (Fig. 4I, K). Both NO_x_ and DIP were higher in the outer CRE (dominated by the CDW) and were lower in regions dominated by the TSW in both seasons (Fig. 4). N/P was generally higher than 50 in the outer CRE in both seasons, and extremely high values exceeding 500 occurred in the southern part of the study area (the mixing region of the CDW and KSSW) in spring where phytoplankton biomass was high (Figs. 4I; 5D, L; S4).

### Distributions of lipid biomarkers and Chl *a*

In spring, the concentrations of brassicasterol ranged from 6.7 to 8184 ng L^–1^ and were highest in the mixing region of the CDW and KSSW, i.e., the river plume front and along the Zhejiang coast (Fig. 5A). Dinosterol concentrations were between 0 and 346 ng L^–1^ and were highest in the outer CRE and south of Hangzhou Bay (both dominated by the CDW) (Fig. 5B). Alkenone concentrations were between 0 and 59 ng L^–1^ and were highest south of Zhoushan Archipelago (Fig. 5C). The sum of all phytoplankton biomarkers measured (∑PB) was between 20 and 8589 ng L^–1^ and had similar distribution patterns to brassicasterol since brassicasterol was the most abundant of the three lipids, being high in the river plume front and along the coast (Fig. 5D). Chl *a* concentrations were between 0.23 and 21.98 μg L^–1^ and were highest in the outer CRE and along the Zhejiang coast (Fig. 5L).

In summer, brassicasterol concentrations were between 42 and 1792 ng L^–1^ and were highest in the regions where the CDW and KSSW mixed well with each other, i.e., river plume front and north and south of the CRE (Fig. 5E). Dinosterol concentrations (range: 6.9–383 ng L^–1^) were highest northeast of the CRE and along the Zhejiang coast (Fig. 5F). C_37_ alkenones (range: 2.1–315 ng L^–1^) were detected at 17 stations and had the highest concentrations outside the CRE and Hangzhou Bay (Fig. 5G). ∑PB (range: 54–1917 ng L^–1^) and Chl *a* (range: 0.05–24.23 μg L^–1^) had similar distributions, showing high concentrations south and north of the Changjiang River plume (Fig. 5H, P).

In both seasons, B/∑PB was highest among the three lipid biomarkers (Table S7) and had high values in the mixing region of the CDW and KSSW, i.e., along the Zhejiang coast in spring and in the north and south off the CRE in summer (Fig. 5I, M). High D/∑PB occurred in the outer CRE and outside Hangzhou Bay in spring and along the Zhejiang coast in summer (Fig. 5J, N). A/∑PB was high in the southern part of the study area in spring and in the southern area of Hangzhou Bay in summer (Fig. 5K, O). The observed seasonal variations in the distribution of phytoplankton biomarkers were broadly correlated with the variation in water masses (Figs. 3, 5). It should be noted that we also evaluated the contribution of each phytoplankton group to total phytoplankton biomass using Chl *a* as the total phytoplankton biomass proxy and we opted not to use those results as lipids and Chl *a* (a pigment) are two different types of phytoplankton biomarkers, with significantly different chemical stability.

## Discussions

### Quantifying the impact of CDW and KSSW on phytoplankton

Three primary surface water masses (the CDW, the TSW, and the KSSW) occurred in the study region during spring and summer (Qi et al. 2014; Zhang et al. 2019; Zhou et al. 2018), with their relative proportions varying as a function of location and season. The spatial extents of the three water masses showed a general northward movement from spring to summer (Fig. 3) (Che and Zhang 2018; Qi et al. 2014; Yang et al. 2013, 2018; Zhou et al. 2018). From spring to summer, the southerly winds and strong river discharge drove the CDW northeastward (Fig. 3A, E), resulting in the main body of CDW occurring to the northeast of the CRE (and outside the study area), with the easternmost extent at 126°E and the northernmost extent north of 33°N (Qi et al. 2014). Consequently, the influence area of CDW in summer was smaller than in spring (Figs. 2, 3). The upwelled KSSW shifted from the surface layer along the Zhejiang coast in spring to the vicinity of the CRE in summer, showing a higher proportion in spring 2017 than that in summer 2018 (Fig. 3C, G). This result is consistent with previous field observations and a physical–biochemical coupled model that showed stronger upwelling in our study region in 2017 compared to 2018 (Luo et al. 2023). Therefore, our results on the distribution and proportions of water masses were consistent with previous findings, suggesting that the three end-member models can be used to evaluate the effects of water masses on phytoplankton ecology.

The changes in phytoplankton biomarker distribution were clearly associated with those in water mass distribution in both seasons, especially for ∑PB, B/∑PB, and D/∑PB with the movement of the CDW and KSSW (Figs. 3, 5). Thus, data from the two seasons were integrated to obtain the responses of lipid biomarkers to water mass changes. Throughout the study area an increase in the proportion of CDW (from 20 to 100%) was associated with a 20% decrease of B/∑PB and a 20% increase of D/∑PB (Fig. 6B, C). In contrast, an increase in the proportion of KSSW (from 0 to 40%) was associated with a 20% increase of B/∑PB and a 20% decrease of D/∑PB (Fig. 6E, F). These results suggested that the CDW favors the predominance of dinoflagellates and the KSSW favors diatom predominance. A similar result was reported based on phytoplankton enumerations in the CRE and adjacent areas in spring 2017 (April 20–May 9 of 2017) (Guo et al. 2020), just before our cruise (May 5–May 19 of 2017), showing that high dinoflagellate abundance occurred outside the CRE and high diatom abundance occurred further seaward. According to phytoplankton abundance in Guo et al. (2020) and per-cell contents of particulate organic carbon in diatoms and dinoflagellates (Finkel et al., 2016), biomass proportions were calculated and showed an ~ 8% increase in diatoms and an ~ 8% decrease in dinoflagellates with increasing salinity (28.4–33.5), consistent with the changes in B/∑PB (9%) and D/∑PB (9%) that accompanied the increase in the proportion of KSSW from 9 to 29% (salinity: 28.3–32.5; Fig. 6E, F). Additionally, most high-∑PB sites (> 1000 ng L^–1^) occurred where the CDW comprised ~ 40–90% of the surface water and KSSW contributed ~ 5–15% (Fig. 6A, D), partially supporting our hypothesis that the interaction of CDW and KSSW promoted total phytoplankton biomass, as well as diatom biomass, but not dinoflagellate biomass.

It should be noted that the mixing of water masses in our study area is very complex and variable, making it difficult to accurately calculate the composition of water masses and the corresponding phytoplankton characteristics. For example, although the contribution of freshwater from the Changjiang River in nearshore waters is significant (e.g., the water volume of 8,964 × 10^8^ m^3^ a^−1^ which is nine times that of all other rivers combined 998 × 10^8^ m^3^ a^−1^ (Qiao et al. 2017)), it is not solely responsible for the low salinity of nearshore waters. The coastal currents from the north, other rivers, and groundwater inputs all have an impact, and there are also significant spatiotemporal changes in the hydrology of such a highly dynamic coastal environment. However, previous studies have shown that the CDW, the TSW, and the KSSW are the main water masses affecting nearshore waters in the ECS (Che et al. 2022; Che and Zhang 2018; Zhou et al. 2018). Our results also show that the calculated proportions of the three water mass from the end-member model were similar to previous results using Nd isotopes, dissolved inorganic iodine species, and dissolved barium (Che et al. 2022; Che and Zhang 2018; Liu et al. 2017; Zhou et al. 2018). Therefore, the consistency between our results and previous findings suggests that our water mass analysis can be used to determine the mean state of water masses and provides a basis to quantify their contributions and influences, while a high spatiotemporal resolution physical–ecological coupling model is still recommended to improve the quantification of water mass characteristics and their impact on phytoplankton distributions.

When applying lipid biomarkers to quantitatively analyze the relationship between phytoplankton community and water mass proportions, one needs to consider the fact that phytoplankton species differ among different water masses at different spatiotemporal scales in our study region (Cen et al. 2017; Chen et al. 2012). Indeed, the production of lipid biomarkers and their response to environmental changes have shown species-specific differences for each phytoplankton group (Bi et al. 2020; Ding et al. 2019; Volkman et al. 1998). For example, certain diatoms have been shown to produce dinosterol, and certain haptophytes, cryptophytes, and dinoflagellates may produce brassicasterol (Giner and Wikfors 2011; Volkman et al. 1998). Not all diatoms produce brassicasterol, and if they do, might only do so in certain conditions (Giner and Wikfors 2011; Volkman et al. 1998). However, previous work on major bloom-forming algae in coastal waters of China showed that dinosterol was detected in all five dinoflagellate species (*Alexandrium minutum*, *Alexandrium tamarense*, *Alexandrium catenella*, *Prorocentrum micans,* and *Scrippsiella trochoidea*) but not in other phytoplankton groups, indicating dinosterol as a reliable biomarker for dinoflagellates in these coastal regions (Geng et al. 2017). Brassicasterol was detected in certain prevailing diatom species (*Cylindrotheca closterium* and *Phaeodactylum tricornutum*), as well as the bloom-forming dinoflagellate *S. trochoidea* and dominant haptophytes such as *Emiliania huxleyi*, in the ECS (Chiang et al. 1999; Ding et al. 2019; Geng et al. 2017; Zhao et al. 2019). It should be noted that POC-normalized contents of brassicasterol in the diatom *P. tricornutum* were up to ~ 20 times higher than those in three dinoflagellates (*Karenia mikimotoi*, *Prorocentrum donghaiense,* and *Prorocentrum minimum*) (Ding et al. 2019). And the abundance of *E. huxleyi* was less than one-tenth of that of diatoms in the ECS (Guo et al. 2014, 2020; Lin et al. 2014; Shih et al. 2019). These findings suggest that diatoms are potential major contributors of brassicasterol in diatom-dominated ecosystems. However, in marine ecosystems where diatoms are not dominant, the contribution of diatoms may be overestimated regarding the use of brassicasterol as a source-specific biomarker for only diatoms. Nevertheless, the observed seasonal variations in the distribution of phytoplankton biomarkers in our study were broadly consistent with previous phytoplankton enumerations (Guo et al. 2014; Zhao et al. 2015), providing good evidence for the applicability of brassicasterol and dinosterol to reveal diatom–dinoflagellate community structure under highly variable environmental conditions. Further studies are recommended to quantify the production of brassicasterol and other source-specific lipids such as highly branched isoprenoid lipids (HBIs) in dominant diatoms to give insights into the more detailed dynamics of phytoplankton communities, and biogeography and ecology of individual species or genera in our study region.

Another consideration is that some large heterotrophic dinoflagellates are capable to consume diatoms and thus play an important role in structuring phytoplankton community composition (Löder et al. 2011; Strom and Buskey 1993). According to previous dilution experiments, in the mesotrophic and oligotrophic waters of marginal seas of China (our study region included), the biomass ratio of heterotrophic dinoflagellates to total microzooplankton was relatively low (mean fraction ratio: ~ 0.1 in the ECS) and showed no significant correlations with Chl *a* concentration (Liu et al. 2021). Thus, heterotrophic dinoflagellates may not be the main factor causing the downward shift of diatoms in our study. Furthermore, there are key features of frustules and forming a colony in certain diatom species, which may reduce predation losses (Kenitz et al. 2020; Petrucciani et al. 2022). In our study region, the dominant diatom species include *C. closterium*, *P. tricornutum* and *Skeletonema costatum* (Guo et al. 2011, 2020; Lin and Li 2017; Tian et al. 2010). They all have the features of frustules and thus have the potential to avoid predation. Considering the ecological efficiency of 10% at the phytoplankton–zooplankton interface (Sommer et al. 2002), we suggest that lipid biomarkers can be applied to reveal phytoplankton community composition in our study region, while the role of predation cannot be completely ruled out and needs to be further studied.

In summary, the influence of water masses on phytoplankton in the Zhejiang coastal region of the western ECS has been inferred by comparing lipid biomarker abundances to water physical and chemical properties. Water masses in the study region had specific temperature, salinity, turbidity, and nutrient characteristics from which three subregions were identified (the CDWR, C-KMR, and KSSWR) (Fig. 3D, H, I) and used to infer the underlying mechanisms of water mass control on phytoplankton.

#### The CDW-dominated region (CDWR)

In the CDWR subregion CDW accounted for > 50% of the surface water and includes the outer CRE and outside Hangzhou Bay (Fig. 3D, H), with quite low KSSW proportions (0–5%). This subregion was characterized by the lowest ∑PB concentrations and B/∑PB, but the highest dinosterol proportions among all subregions (Fig. 5; Table S8). As the CDW proportion increased from ~ 50% to ~ 100%, there was a distinct decrease of ∑PB concentrations, a 10% decrease of brassicasterol proportions, and a 10% increase of dinosterol proportions (Fig. 6A, B, C), indicative of a strong decrease in phytoplankton biomass and diatom proportions, and an increase in dinoflagellate proportions.

The low phytoplankton biomass in the CDWR subregion can be attributed to light limitation induced by high turbidity of the CDW, with the phytoplankton community structure additionally regulated by nutrient ratios (Jiang et al. 2014; Wang et al. 2016a). Low photosynthetically active radiation (PAR) caused by high turbidity where high proportions of CDW occurred likely reduced phytoplankton biomass (Tseng et al. 2014), as suggested by the negative correlations between ∑PB concentrations and CDW proportions (and TSM concentrations) in the RDA results (Fig. 7A) and high phytoplankton biomass generally occurred outside the CRE where TSM concentrations were low (Fig. S5) (Tseng et al. 2014; Wang et al. 2016a). Where the CDW proportions were less than ~ 50%, the inhibiting effect of the CDW with high turbidity on ∑PB concentrations and B/∑PB disappeared (Fig. 6A). Therefore, variations in the proportion of CDW may explain the differential effects of CDW on phytoplankton biomass in previous studies, such as the negative effects in the inner estuary and positive effects in the adjacent waters (Chai et al. 2006). Moreover, N/P in surface waters was high (> 50) in the CRE and adjacent areas both in spring and summer (Fig. 4I, K), which likely contributed to the predominance of dinoflagellates in the CDWR subregion (Bi et al. 2021; Huang et al. 2005; Jiang et al. 2014; Ou et al. 2015). Certain traits of dinoflagellates may contribute to the relatively high biomass of dinoflagellates under high N/P conditions, such as the ability of heterotrophic and mixotrophic dinoflagellates to utilize dissolved or particulate organic phosphorus and to ingest dissolved organic matter (produced after the early spring algae bloom in the CDWR subregion) (Glibert and Burford 2017; Li et al. 2009).

#### The CDW–KSSW mixing region (C-KMR)

The C-KMR subregion was characterized by CDW proportions of 40–90% and KSSW proportions of 5%–15% that occurred outside the CDWR subregion and along the Zhejiang coast. It had the highest ∑PB (Table S8), and by extension the highest phytoplankton biomass in our study area. The C-KMR subregion occurred near the outcrops of strong upwelling and moved northward from spring to summer (Fig. 3D, H).

Compared to the CDWR subregion, lower CDW proportions and higher KSSW proportions in the C-KMR subregion resulted in less light limitation (associated with low TSM concentrations) and greater concentrations of both DIN and DIP (Figs. 4, S5). DIP supplied by CDW and KSSW was the most important factor controlling phytoplankton biomass and community structure in this subregion (Fig. 7B). Under such high-nutrient conditions, diatom proportions inferred from B/∑PB significantly increased (~ 20%) with increasing KSSW proportions (from 0 to 40%) and decreasing CDW proportions (from 100 to 15%) (Fig. 6B, E), aligning with phytoplankton ecological traits, such as higher nutrient requirements of diatoms (*r*-strategy) compared to dinoflagellates (*K*-strategy) (Margalef 1978). As a result, ∑PB concentrations and B/∑PB in the C-KMR subregion were higher than those in the CDWR subregion (Fig. 6; Table S8). Consistent with this observation, previous studies also showed that in summer the upwelling together with the CDW provided abundant nutrients for the high biomass of diatoms in the vicinity of CRE (Liu et al. 2016). Pigment analyses by Xu et al. (2019) showed that diatoms outcompete dinoflagellates in the high-nutrient (especially DIP) water under the influence of CDW and KSSW. High total phytoplankton biomass and diatom proportions in the C-KMR subregion are therefore attributable to the joint effects of the CDW and the KSSW, with high DIP from the KSSW being particularly important.

#### The KSSW-dominated Region (KSSWR)

The KSSWR subregion was characterized by a > 20% proportion of KSSW and a < 60% proportion of CDW. It was located slightly offshore and adjacent to upwelling outcrops (Fig. 3). Where the proportion of CDW decreased from ~ 50% to 20% (and salinity increased from ~ 29 to 33), ∑PB concentrations were halved (from ~ 1000 ng L^–1^ to ~ 500 ng L^–1^; Fig. 6A). This observation is consistent with those from the northeastern ECS where ∑PB declined from ~ 1500 ng L^–1^ to < 500 ng L^–1^ as salinity increased from ~ 30 to ~ 34 (Bi et al. 2018). Compared to the C-KMR subregion the KSSWR subregion had lower ∑PB concentrations and slightly higher B/∑PB (Table S8).

The contribution of CDW in the KSSWR subregion was lower than in the CDWR and C-KMR subregions (Table S8). The CDW is an important source of nutrients to the mid- and outer shelf of the ECS (Figs. 3, 4) (Bi et al. 2018; Kim et al. 2009), characterized by low salinity and high concentrations of nutrients especially DIN, and its contribution decreases seaward (Che and Zhang 2018; Kim et al. 2009; Li et al. 2014b). Previous studies reported negative correlations between lipid biomarkers and salinity (Bi et al. 2018; Kim et al. 2016) and positive correlations between lipid biomarkers and nutrient concentrations (Bi et al. 2018) in the ECS and adjacent areas. Thus, less CDW was likely responsible for reduced lipid biomarker concentrations along the CDW transport pathway seaward. With the the continuous offshore decrease of the CDW proportion and increase of open-sea water, DIN is likely to limit phytoplankton growth especially when NO_x_ concentrations are near the half-saturated concentration of 1.0 μmol L^−1^ (Figs. 4A-D; 7) (Chen et al. 2001; Eppley et al. 1969; Van Oostende et al. 2017). The lower proportion of high-DIN CDW in the KSSWR subregion might thereby explain its lower phytoplankton biomass than in the C-KMR subregion, while the interaction of the CDW and the KSSW yielded a similar phytoplankton community within the KSSWR and C-KMR subregions (Table S8).

### Implications on marine ecology and environmental management

**Fig. 8:**
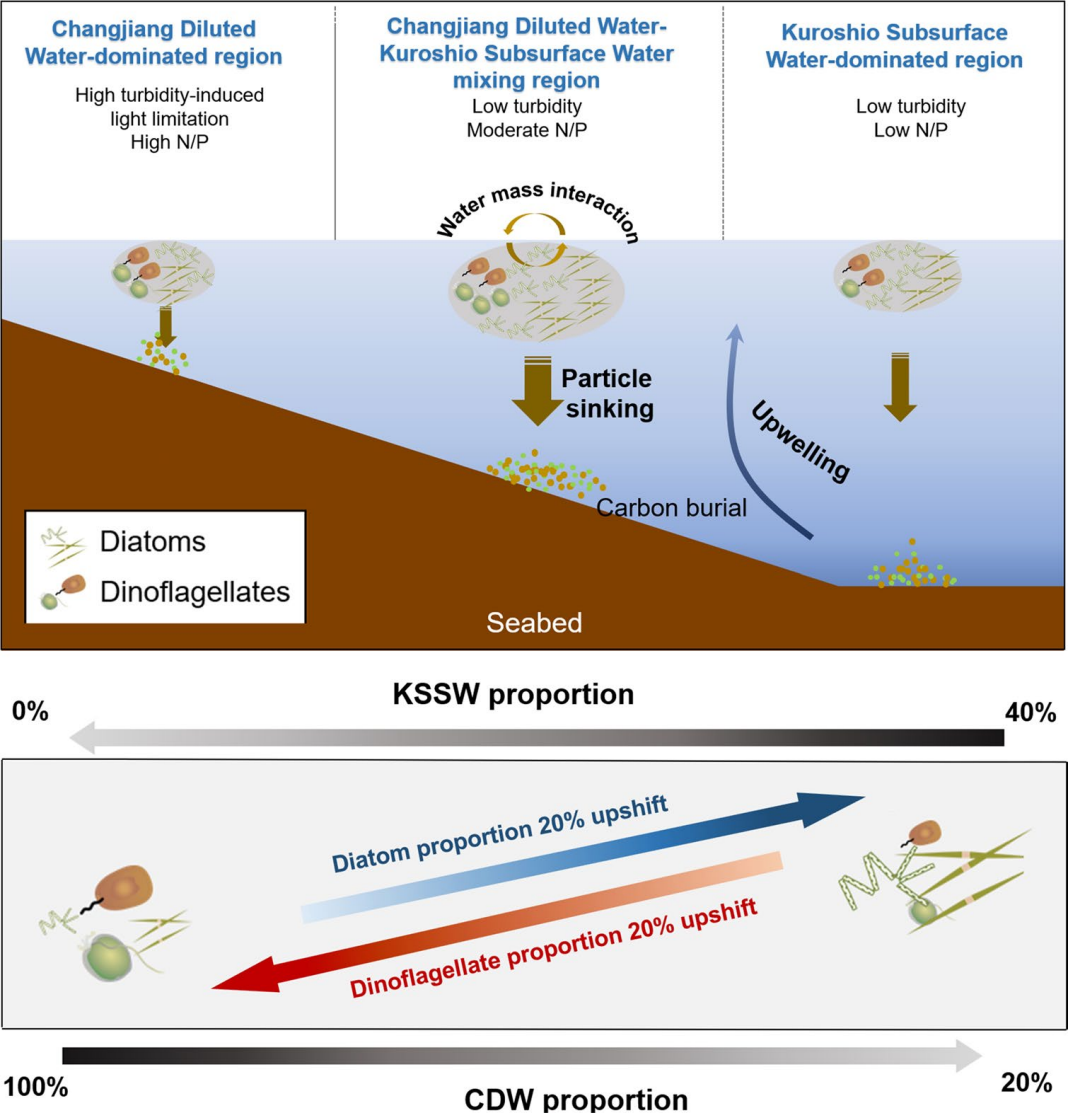
Conceptual illustration of the control of phytoplankton community structure in surface water and particle export in Zhejiang coastal waters in spring and summer. Brown circles with different diameters represent total biomass of diatoms and dinoflagellates. The width of arrows in the upper panel represents the magnitude of particulate organic carbon flux. It should be noted that this figure shows the relative changes in the biomass of diatoms and dinoflagellates. For water mass abbreviations refer to the caption of Fig. 2

The three subregions identified in this study had distinct hydrographic and nutrient characteristics that resulted in differing phytoplankton biomass and community structure (Table S8; Fig. 8). Here we specifically evaluate the relative changes in the biomass of diatoms and dinoflagellates caused by the influence of water mass proportion changes in spring and summer. Overall, total biomass of diatoms and dinoflagellates was higher in the C-KMR subregion than other two subregions, with a higher biomass proportion of dinoflagellates in the CDWR subregion and that of diatoms in other two subregions. These results are consistent with microscopy and photosynthetic pigment observations in our study region, which have shown clear differences in diatom–dinoflagellate community composition in different water masses (Liu et al. 2016).

As a large river-dominated region, the CDWR subregion is known to have frequent harmful algal blooms (HABs) often dominated by dinoflagellates such as *K. mikimotoi* (Li et al. 2014a, b; Zhou et al. 2022). Our results suggest that reducing DIN concentrations in the CDW may reduce the proportion of dinoflagellates and lessen the occurrence of HABs in the CDWR subregion, but not necessarily in the C-KMR and the KSSWR subregions which are more strongly influenced by oceanic water masses. Previous studies have shown that a reduction of DIP without a simultaneous decrease of DIN is not sufficient to limit eutrophication in the freshwater–marine continuum, because large amounts of DIN along with regenerated DIP and dissolved silicate can still increase phytoplankton biomass (Ratmaya et al. 2019). Accordingly, the Chinese government has controlled dual nutrient (DIN and DIP) input to the watershed in the past decades. For example, total phosphorus (particulate and dissolved) concentrations at the Datong hydrological station (downstream of the Changjiang River) were reduced by 52% from 1980 to 2015 (Hu et al. 2020). In addition, total nitrogen (organic and inorganic) input from the Taihu watershed (downstream of the Changjiang River) was also reduced by 17% from 2010 to 2015 (Wu et al. 2022). Based on our results and previous findings, it can thus be speculated that with the reduction of DIN concentrations and N/P ratios in the CDWR subregion, toxic dinoflagellates such as *K. mikimotoi* may have been outcompeted by nontoxic *P. donghaiense*, because the competitive advantage of *P. donghaiense* is greater than *K. mikimotoi* as N/P ratios decrease (Li et al. 2009). This succession of phytoplankton composition from toxic to nontoxic dinoflagellates would potentially benefit natural fisheries along the ECS, such as the Zhoushan fishing ground.

Outside the CDWR subregion, the CDW significantly augments phytoplankton biomass on the ECS shelf (Bi et al. 2018; Wang et al. 2022; Wu et al. 2016). With the CDW proportion decreasing from 69% (in the C-KMR subregion) to 51% (in the KSSWR subregion), 10% (in Tsushima region), and even negligible (in the Kuroshio region), ∑PB decreased from 949 ± 838 ng L^–1^ to 704 ± 504 ng L^–1^, 89 ± 26 ng L^–1^, and 51 ± 26 ng L^–1^, respectively (Che and Zhang 2018; Wang et al. 2022) (Fig. S6). It is therefore expected that management of runoff and nutrient fluxes into the CDW could influence phytoplankton biomass on the ECS shelf, as the ∑PB concentration reduced by an average of 136 ± 119 ng L^–1^ with the CDW proportion reducing by 10% (Fig. S6).

High phytoplankton biomass and diatom proportions in the C-KMR subregion have important implications for the marine carbon cycle (Fig. 8). Phytoplankton lipid input flux is defined as the product of lipid concentrations and sinking rates of phytoplankton, and the average deposition rate of six common diatom species is about 2.6 times that of six dinoflagellate species in the ECS (0.95 m day^–1^ for diatoms and 0.37 m day^–1^ for dinoflagellates) (Cao et al. 2022; Guo et al. 2016). Recently, the proto-burial efficiency of ∑PB has been quantified in the eastern China marginal seas, showing overall similar values and spatial patterns to that of marine organic carbon (Cao et al. 2022). Based on sinking rates of diatoms and dinoflagellates and the concentrations of brassicasterol and dinosterol, we estimated the lipid biomarker input flux in the three subregions (54.8 kg km^−2^ a^−1^ in the CDWR subregion, 153 kg km^−2^ a^−1^ in the C-KMR subregion, and 114 kg km^−2^ a^−1^ in the KSSWR subregion) and the entire study area (102 kg km^−2^ a^−1^) (Text S1; Table S8). Specifically, integrated data from previous studies in 2011, 2013, and 2015 (Cao et al. 2022; Chen et al. 2017; Wang et al. 2019a, b; Wu et al. 2016) and this study show that the C-KMR subregion lipid input flux (average: 304 kg km^−2^ a^−1^) is 2.3 and 3.1 times higher than that in the KSSWR subregion (average: 134 kg km^−2^ a^−1^) and in the CDWR subregion (average: 97 kg km^−2^ a^−1^), respectively, highlighting the significance of the C-KMR subregion in the marine carbon cycle of the western ECS (Fig. S7).

## Conclusions

This study combined hydrological and nutrient data with phytoplankton lipid biomarkers to partition and quantify riverine and oceanic controls on phytoplankton biomass and community structure in the highly dynamic Zhejiang coast of the ECS. The distribution and composition of lipid biomarkers varied with water mass proportions and showed a clear seasonal variation, with a northward shift of high brassicasterol (mainly from diatoms) and ∑PB from spring to summer. The increase in the proportion of CDW from 20 to 100% was associated with a 20% decrease in brassicasterol proportions and a 20% increase in dinosterol proportions. In contrast, an increase in the fraction of KSSW from 0 to 40% was associated with a 20% increase in brassicasterol proportions and a 20% decrease of dinosterol proportions. The CDW-dominated region was characterized by the highest proportion of dinoflagellates and the lowest total phytoplankton biomass, while the CDW–KSSW mixing region had the highest phytoplankton biomass and diatom predominance. The KSSW-dominated region had a moderate total phytoplankton biomass and an overall similar phytoplankton community with the CDW–KSSW mixing region. The regulation of CDW and KSSW on phytoplankton biomass and community structure was a result of the combined effects of turbidity and nutrient composition. Given the apparent differences in phytoplankton biomass and community structure in regions of the ECS that are impacted by different water masses, our results suggest that management of nutrient loading to the coastal zone can result in marked and predictable changes in the phytoplankton community in the western ECS with socioeconomic benefits. Further studies within individual seasons are required to better understand the phytoplankton response to water mass variations.

## Supplementary Information

Below is the link to the electronic supplementary material.

Supplementary file1 (DOC 11382 KB)

## Data Availability

Data of all Figures in this paper are available from the Supporting Information and Gao et al., (2023) https://doi.org/10.5281/zenodo.10074848.
