# Supplementary material for: Assessing the interaction of oceanic and riverine processes on coastal phytoplankton dynamics in the East China Sea
Source: Mar Life Sci Technol. 2025 Jan 7;7(1):157–75. doi: 10.1007/s42995-024-00260-y (PMC11871228; doi:10.1007/s42995-024-00260-y)
Supplement: Supplementary file 1 — Supplementary file1 (DOC 11382 KB) [file 42995_2024_260_MOESM1_ESM.doc]

Supporting Information for

**Assessing the interaction of oceanic and riverine processes on coastal phytoplankton dynamics in the East China Sea**

Jiawei Gao ^1,2^, Rong Bi ^1,2^, Julian P. Sachs ^1,3^, Yaoyao Wang ^1,2^, Yang Ding^1,2^, Hong Che ^2^, Jing Zhang ^1,4^, Peng Yao ^1,2^, Jie Shi ^2,5^, and Meixun Zhao ^1,2^

^1^ Frontiers Science Center for Deep Ocean Multispheres and Earth System, and Key Laboratory of Marine Chemistry Theory and Technology, Ministry of Education, Ocean University of China, Qingdao 266100, China

^2^ Laoshan Laboratory, Qingdao 266237, China

^3^ School of Oceanography, University of Washington, Seattle, WA, United States

^4^ Faculty of Science, Academic Assembly, University of Toyama, Toyama 9308555, Japan

^5^ Key Laboratory of Marine Environment and Ecology, Ministry of Education of China, Ocean University of China, Qingdao 266100, China

Corresponding author: Rong Bi (rongbi@ouc.edu.cn)

**Introduction**

Supporting information includes text (Text S1), seven figures (Figures S1 to S7) and seven tables (Tables S1 to S8). Text 1 shows the detailed calculation of the phytoplankton lipid input flux mentioned in the section 4.3 in main text. Besides, all data in figures and tables of the Supporting Information have been introduced in the section Materials and Methods in main text.

Temperature, salinity and chlorophyll *a* (Chl *a*) were measured in situ using CTD instruments (SBE 25, Sea Bird Electronics Inc., USA) during cruise NORC2017–03, RBR concerto logger (RBR Ltd., Ottawa, Canada) during MZ17SP and RBR XR–620 (RBR Ltd., Ottawa, Canada) during MZ18SU, respectively. Suspended particle samples for lipid biomarker analyses and TSM (total suspended matter) were collected from surface seawaters (depth: 3 m; water volume: 10–50 L) for 61 and 57 stations in spring and summer, respectively (Table S1). Water samples for nutrient measurements were collected through cellulose acetate membranes for 37 and 57 stations in spring and summer, respectively, at the depths similar to those of suspended particle samples (Table S1).

Water masses proportions in the bottom layer were calculated based on end-member values for the Changjiang Diluted Water (CDW), the Kuroshio Subsurface Water (KSSW), and the Taiwan Strait Water (TSW) (Table S3) using a Bayesian Markov chain Monte Carlo method and the MATLAB package. The proportions of each water mass present at each sample location were then used to predict the initial nutrient concentrations (NO_x_ and DIP) in the surface and bottom waters that would result from conservative mixing of the water masses based on nutrient concentrations in the three end-members, with strongly significant positive correlation existing between the initial and measured concentrations of NO_x_ and DIP (Fig. S1; S2). Spearman’s correlation analysis was conducted to quantitatively assess the relationship between nutrient concentrations (in-situ measured and model-derived values) and Chl *a* concentrations in both seasons using IBM SPSS 25 software.

The similarity of ∑PB, B/∑PB, D/∑PB and A/∑PB between the three subregions (the CDW-dominated region, the CDW-KSSW mixing region and the KSSW-dominated region) was tested with a Kruskal-Wallis rank sum test (Table S6). Prior to the similarity test, data were tested for normality and homogeneity of variances via a Shapiro-Wilk test and a Levene's test, respectively, in the three subregions (Table S5).

Text S1.

Furmulas used to estimate the lipid input flux in the CDWR, the C-KMR, the KSSWR subregions and the whole study area.


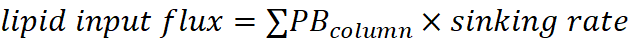


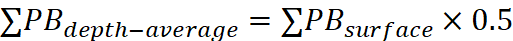


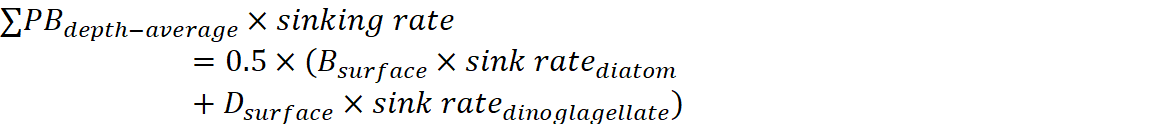


where ∑PB (the sum of brassicasterol, dinosterol, and C_37_ alkenones) is total lipid biomarkers (considering only brassicasterol and dinosterol due to their high concentrations). Depth-averaged phytoplankton biomass were estimated as half of the phytoplankton biomass in surface suspended particles (Cao et al. 2022; Guo et al. 2016; Jiang et al. 2015). B and D represent brassicasterol and dinosterol, respectively. The CDWR, the C-KMR and the KSSWR represent the Changjiang Diluted Water-dominated region, the Changjiang Diluted Water-Kuroshio Subsurface Water Mixing Region and the Kuroshio Subsurface Water-dominated region, respectively. According to formulas above, the lipid input flux in the CDWR, the C-KMR, the KSSWR subregions and the whole region are 54.8, 153.0, 113.8 and 101.8 kg km^−2^ yr^−1^, respectively.


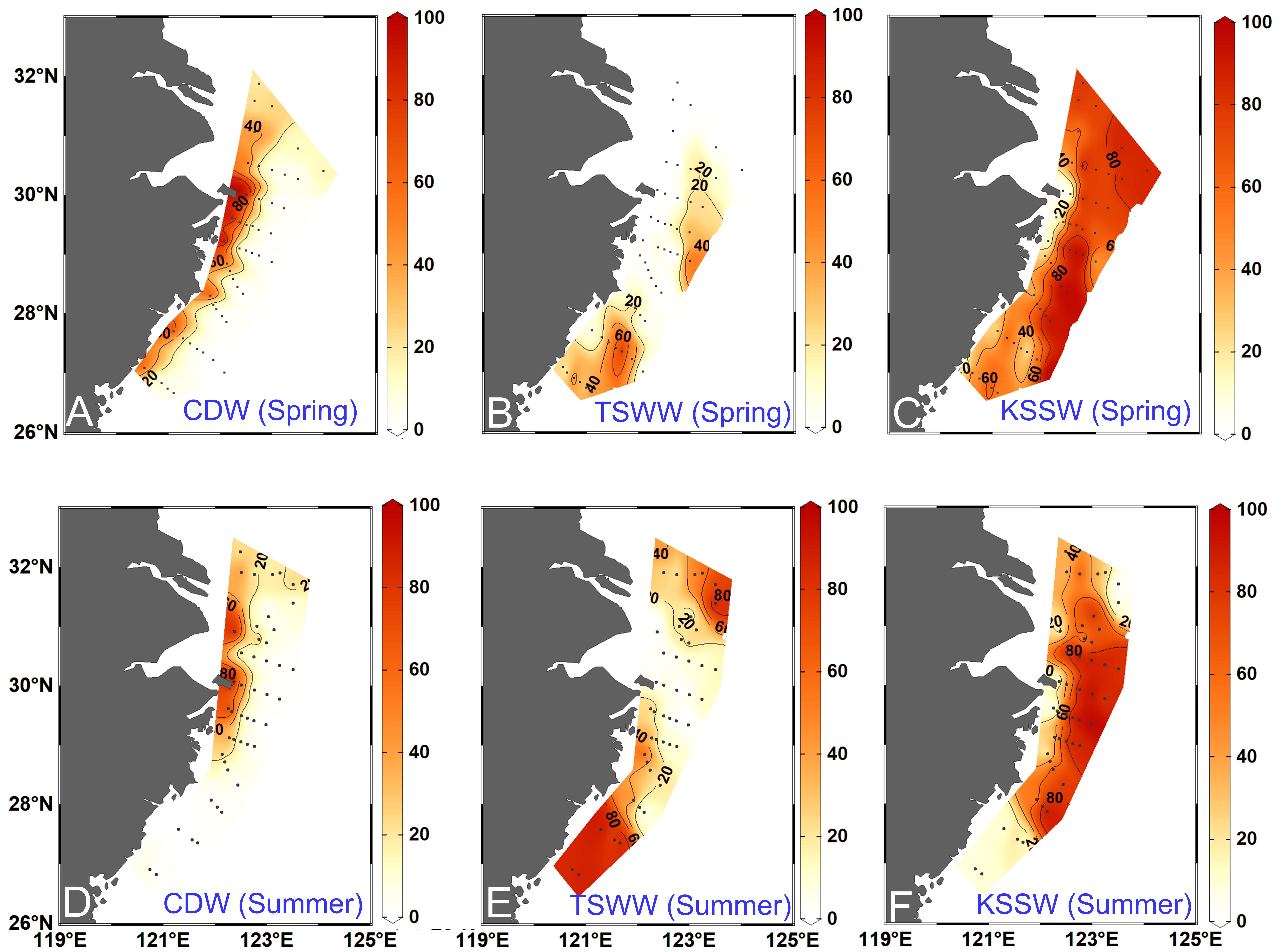


**Fig. S1** Proportions (%) of three water masses in the bottom water at each sampling station in spring 2017 and summer 2018. CDW: Changjiang Diluted Water; KSSW: Kuroshio Subsurface Water; TSW: Taiwan Strait Water. Data from 52 and 46 stations were used in spring and summer, respectively, and the remaining stations were outside the range of end-members and thus excluded


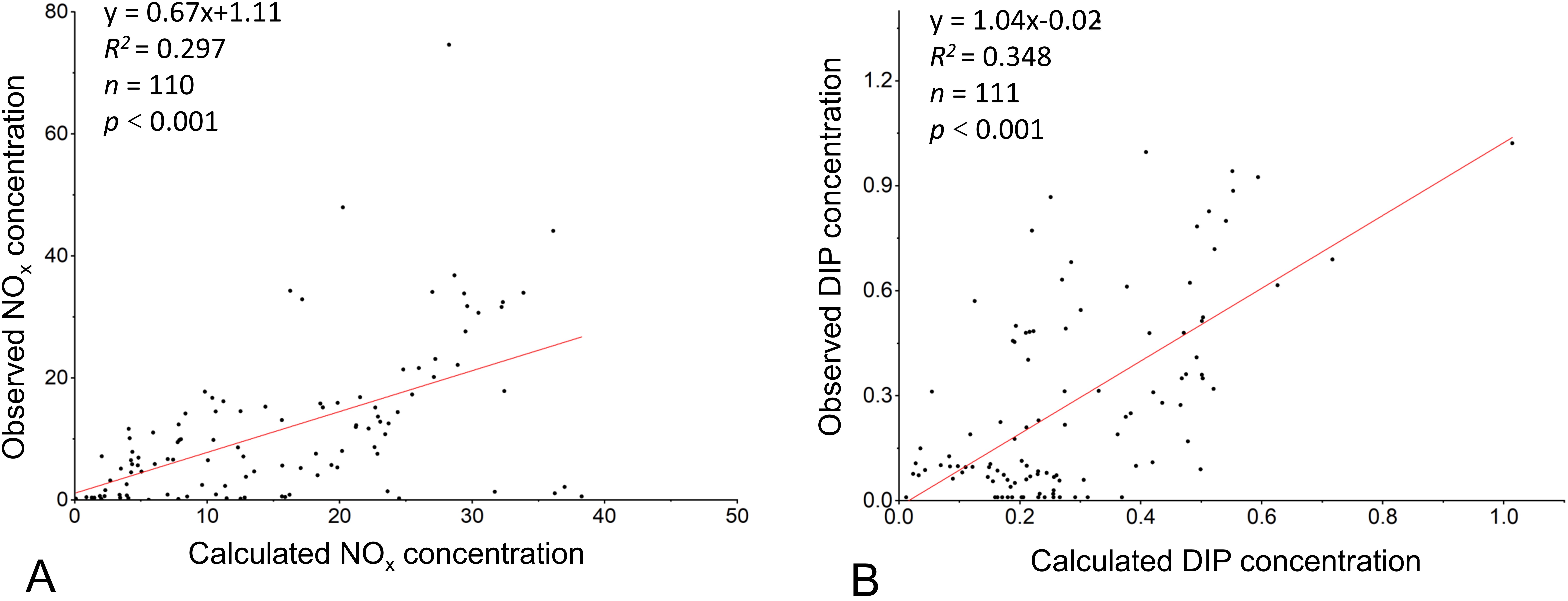


**Fig. S2** Relationships between the observed and calculated nutrient concentrations (μmol L^−1^). Panels A and B include samples from the surface and bottom layers





**Fig. S3** Vertical distributions of temperature (℃; B, D, F and H) and salinity (C, E, G and I) along the transects of CRE and f in spring 2017 and summer 2018


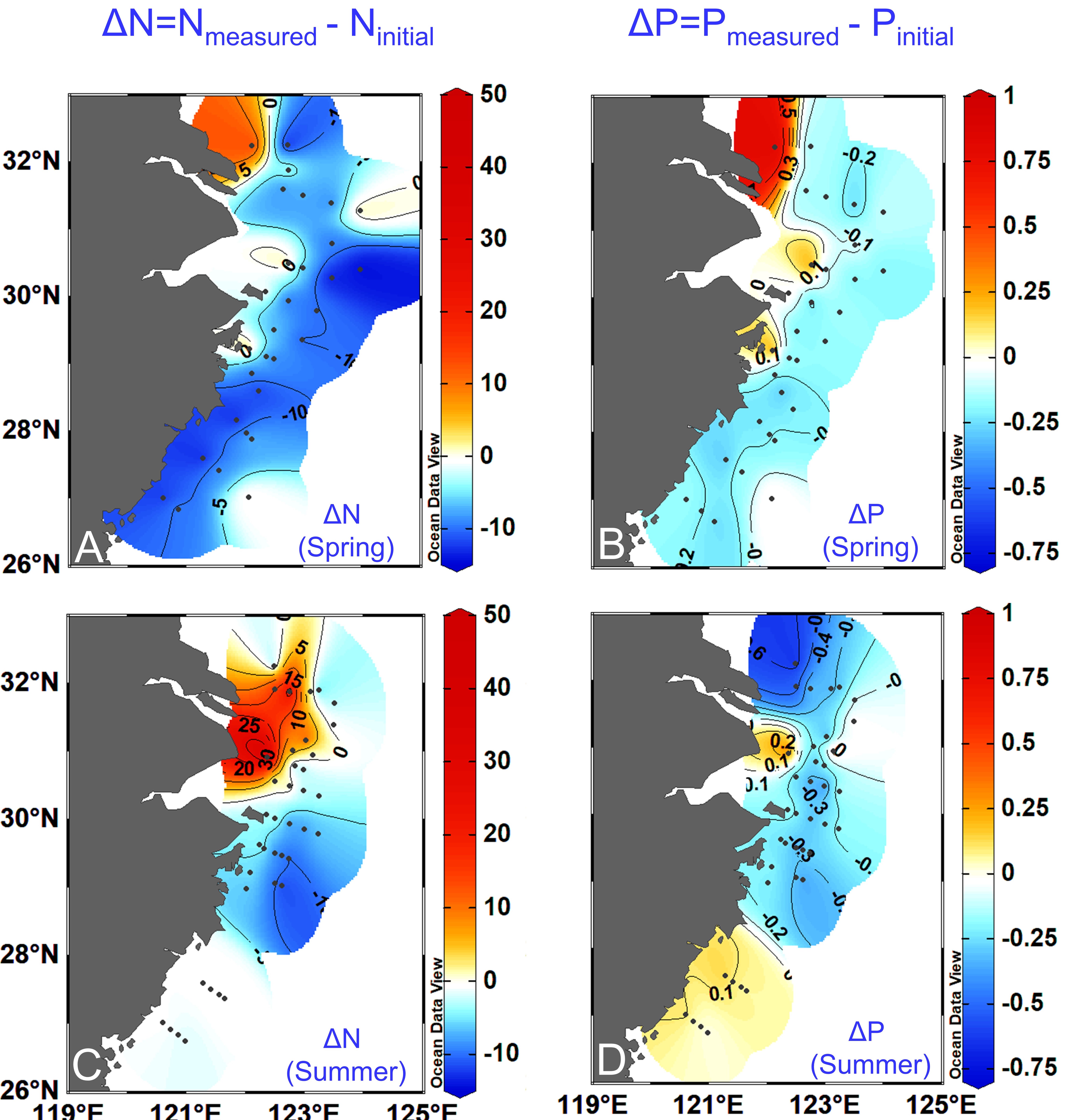


**Fig. S4** Distributions of (A and C) ΔN = N_measured_ – N_initial_ (the differences between field-measured and estimated initial values of NOx (NO_2_^–^+NO_3_^–^), μmol L^–1^) and (B and D) ΔP = P_measured_ – P_initial_ (the differences between field-measured and estimated initial values of DIP (phosphate), μmol L^–1^) in the surface water in spring 2017 and summer 2018. The negative values of ΔN and ΔP imply nutrient consumption by phytoplankton, and the positive values imply nutrient production process by organic matter mineralization. Note that both ΔN and ΔP at stations closer to the Changjiang River Estuary than the station b1 (the end-member of CDW) were generally higher than zero in both seasons, due to the higher nutrient concentrations than B1


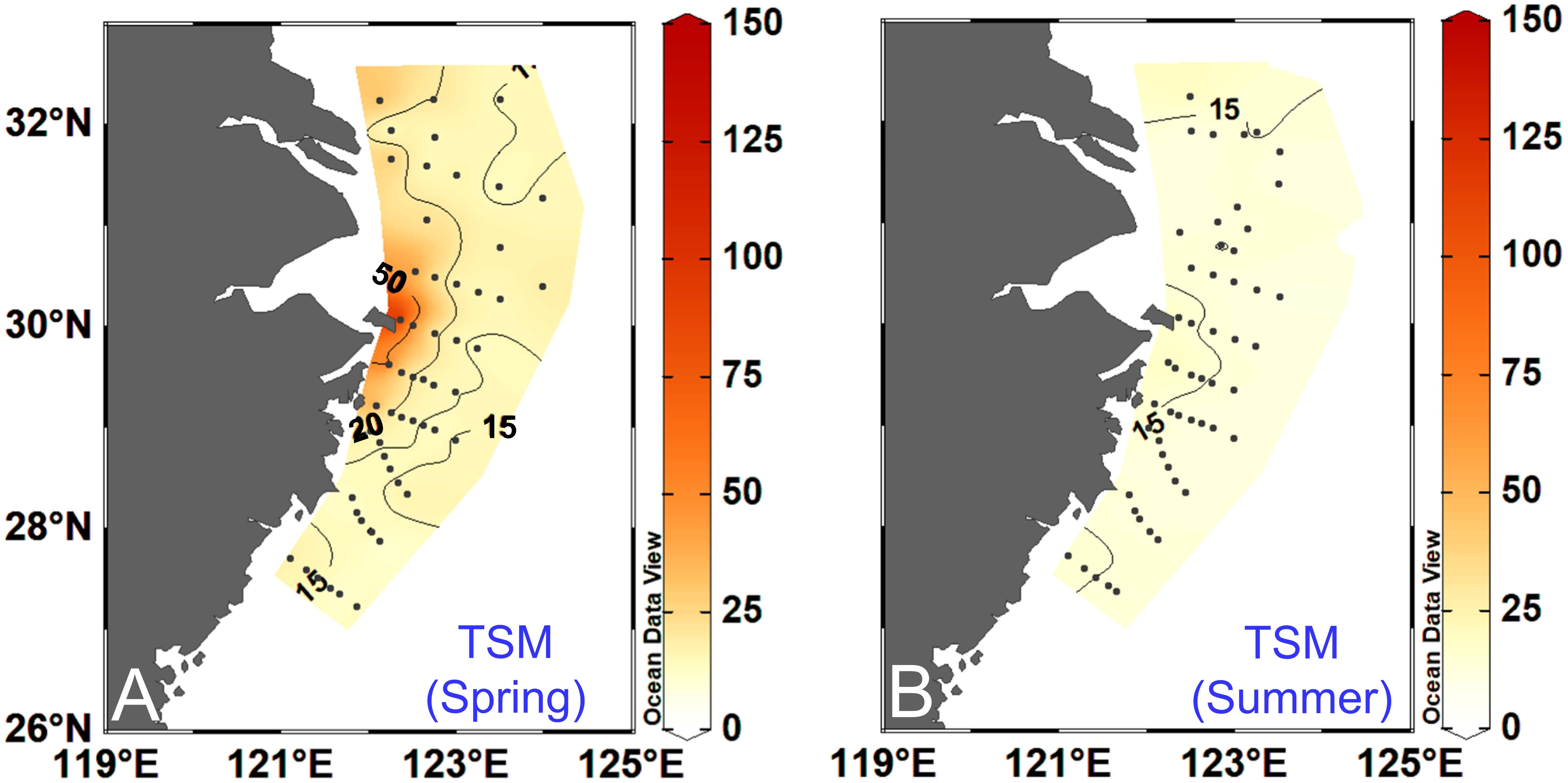


**Fig. S5** Distributions of TSM (suspended particulate matter, mg L^−1^) in the surface layer in spring 2017 and summer 2018. TSM in spring (panel A) is higher in the outer CRE and the Hangzhou Bay where the CDW proportion is high, while TSM in summer (panel B) is low in those regions


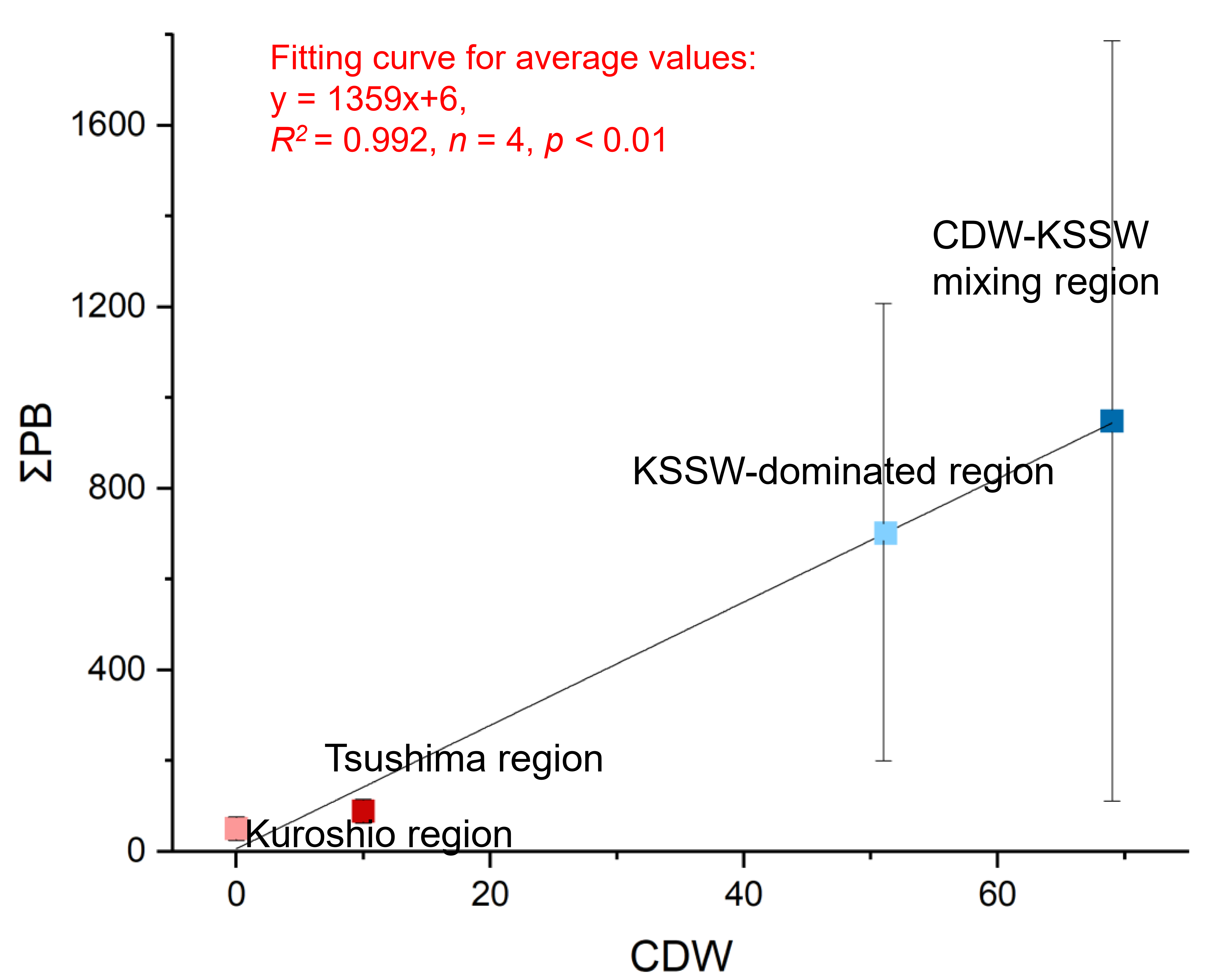


**Fig. S6** Relationship between average values of the Changjiang Diluted Water proportion (CDW, %) and average values of ∑PB (ng L^–1^, ±SD) in the CDW-KSSW mixing region (this study), KSSW-dominated region (this study), Kuroshio region and Tsushima region (Che & Zhang, 2018; Wang et al., 2022)


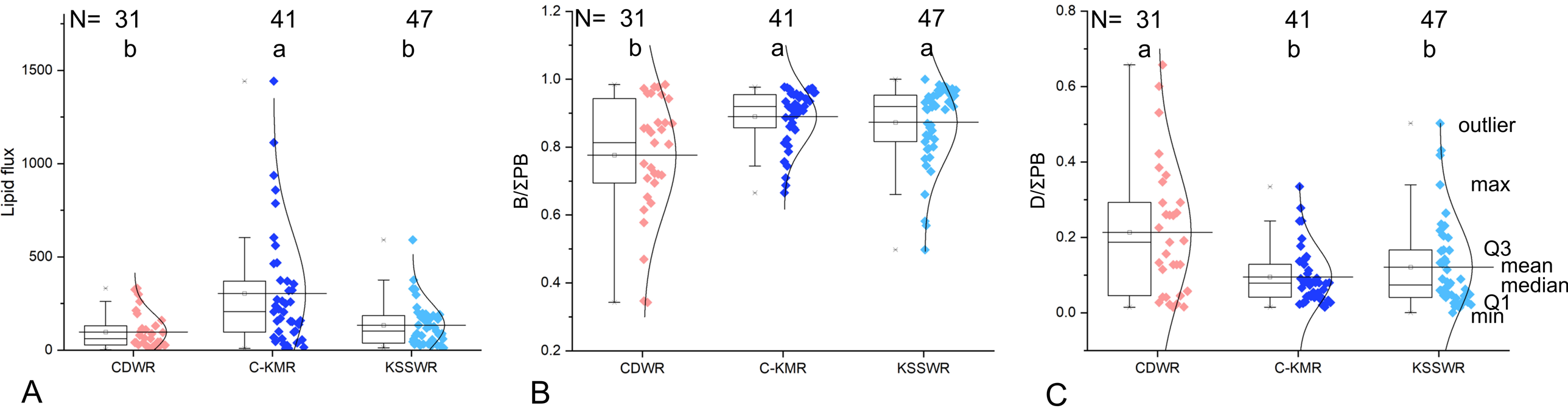


**Fig. S7** Box plot of lipid flux (kg km^−2^ yr^−1^), B/∑PB and D/∑PB of integrated data in the CDW-dominated region (CDWR), the CDW-KSSW mixed region (C-KMR) and the KSSW-dominated region (KSSWR). The letters a and b in the panels denote significant difference between different subregions. Data are obtained from previous studies (Cao et al., 2022; Chen et al., 2017; Wang et al., 2019; Wu et al., 2016) and this study

**Table S1** Summary of sample information and the data of lipid biomarker concentrations (∑PB), temperature, salinity, chlorophyll *a* and nutrient concentrations in the surface layer in spring 2017 and summer 2018. Water volume refers to the sampling water volume for lipid analysis. ∑PB: brassicasterol + dinosterol + C_37_ alkenones. For station names, labels with “-X” (X = 1~11) represent specific sampling locations but not dual sampling from transect a during the spring cruise NORC2017-03 and summer cruise MZ18SU.

| Season | Station | Sampling date | Longitude | Latitude | Depth | Sampling depth | Temperature | Salinity | Chlorophyll *a* | Water volume | ∑PB | Brassicasterol | Dinosterol | C_37_ alkenones | DIP | NO_x_ |
| --- | --- | --- | --- | --- | --- | --- | --- | --- | --- | --- | --- | --- | --- | --- | --- | --- |
|  | name | Day/month | (E/°) | (N/°) | (m) | (m) | (℃) |  | (μg L^−1^) | (L) | (ng L^−1^) | (ng L^−1^) | (ng L^−1^) | (ng L^−1^) | (μmol L^−1^) | (μmol L^−1^) |
| Spring | a1^a^ | 10/5 | 122.53 | 30.54 | 27 | 3 | 18.42 | 26.92 | 1.00 | 25 | 138 | 88 | 51 | 0 | ND | ND |
| Spring | a2^a^ | 10/5 | 122.75 | 30.49 | 41.4 | 3 | 18.23 | 27.13 | 1.98 | 25 | 20 | 6.7 | 13 | 0 | 0.48 | 33.85 |
| Spring | a3^a^ | 11/5 | 123.00 | 30.42 | 52.4 | 3 | 18.86 | 28.34 | 1.95 | 25 | 432 | 312 | 112 | 8 | 0.04 | 17.3 |
| Spring | a4^a^ | 11/5 | 123.25 | 30.34 | 58.6 | 3 | 19.32 | 29.95 | 0.46 | 25 | 372 | 340 | 30 | 2.8 | ND | ND |
| Spring | a5^a^ | 11/5 | 123.50 | 30.27 | 67.2 | 3 | 19.54 | 30.20 | 0.23 | 25 | 206 | 183 | 19 | 4.5 | 0.01 | 4.04 |
| Spring | b1^a^ | 10/5 | 122.36 | 30.07 | 16.2 | 3 | 17.67 | 26.85 | 0.55 | 10 | 150 | 87 | 63 | 0 | 0.23 | 32.43 |
| Spring | b2^a^ | 10/5 | 122.50 | 30.01 | 22.6 | 3 | 18.26 | 27.43 | 1.71 | 25 | 114 | 74 | 40 | 0 | ND | ND |
| Spring | b3^a^ | 10/5 | 122.75 | 29.93 | 42.6 | 3 | 18.72 | 29.41 | 2.70 | 25 | 618 | 450 | 163 | 4.7 | 0.02 | 11.72 |
| Spring | b4^a^ | 10/5 | 123.00 | 29.86 | 55.6 | 3 | 19.74 | 28.08 | 0.78 | 25 | 316 | 227 | 81 | 6.9 | ND | ND |
| Spring | b5^a^ | 10/5 | 123.24 | 29.78 | 61.5 | 3 | 20.13 | 30.74 | 1.55 | 25 | 1908 | 1832 | 63 | 13 | 0.01 | 5.64 |
| Spring | c1^a^ | 8/5 | 122.23 | 29.62 | 22 | 3 | 17.26 | 27.81 | 0.85 | 10 | 146 | 102 | 44 | 0 | 0.59 | 34.42 |
| Spring | c2^a^ | 9/5 | 122.37 | 29.54 | 17.2 | 3 | 17.79 | 28.26 | 0.53 | 25 | 387 | 319 | 64 | 4.2 | ND | ND |
| Spring | c3^a^ | 9/5 | 122.51 | 29.50 | 29.8 | 3 | 18.29 | 28.15 | 3.09 | 25 | 1117 | 793 | 310 | 14 | 0.02 | 20.17 |
| Spring | c4^a^ | 9/5 | 122.62 | 29.48 | 45 | 3 | 18.79 | 28.45 | 1.80 | 25 | 700 | 596 | 89 | 14 | ND | ND |
| Spring | c5^a^ | 9/5 | 122.74 | 29.42 | 54.8 | 3 | 18.78 | 29.77 | 1.88 | 25 | 663 | 563 | 76 | 25 | ND | ND |
| Spring | c6^a^ | 9/5 | 122.99 | 29.35 | 61.2 | 3 | 19.66 | 30.19 | 1.69 | 25 | 2228 | 2096 | 92 | 40 | 0.01 | 7.6 |
| Spring | d1^a^ | 8/5 | 122.08 | 29.22 | 8 | 3 | 18.80 | 27.39 | 0.82 | 10 | 258 | 246 | 12 | 0 | 0.5 | 34.1 |
| Spring | d2^a^ | 8/5 | 122.25 | 29.15 | 17.1 | 3 | 18.70 | 28.56 | 5.86 | 25 | 2108 | 2026 | 57 | 25 | ND | ND |
| Spring | d3^a^ | 8/5 | 122.37 | 29.10 | 34.6 | 3 | 17.91 | 27.78 | 7.32 | 25 | 2766 | 2682 | 57 | 27 | 0.03 | 22.15 |

**Table S1** continued

| Season | Station | Sampling date | Longitude | Latitude | Depth | Sampling Depth | Temperature | Salinity | Chlorophyll *a* | Water volume | ∑PB | Brassicasterol | Dinosterol | C_37_ alkenones | DIP | | NO_x_ |
| --- | --- | --- | --- | --- | --- | --- | --- | --- | --- | --- | --- | --- | --- | --- | --- | --- | --- |
|  | name | Day/month | (E/°) | (N/°) | (m) | (m) | (℃) |  | (μg L^−1^) | (L) | (ng L^−1^) | (ng L^−1^) | (ng L^−1^) | (ng L^−1^) | (μmol L^−1^) | | (μmol L^−1^) |
| Spring | d4^a^ | 8/5 | 122.50 | 29.07 | 49.8 | 3 | 18.61 | 29.02 | 2.66 | 25 | 8589 | 8184 | 346 | 59 | 0.08 | | 12.56 |
| Spring | d5^a^ | 8/5 | 122.62 | 29.03 | 57.2 | 3 | 18.52 | 29.67 | 1.31 | 25 | 3493 | 3368 | 109 | 16 | ND | | ND |
| Spring | d6^a^ | 8/5 | 122.75 | 28.98 | 60.4 | 3 | 18.82 | 29.82 | 4.12 | 25 | 1009 | 941 | 45 | 22 | ND | ND | |
| Spring | d7^a^ | 8/5 | 122.99 | 28.87 | 67 | 3 | 21.54 | 33.41 | 0.42 | 25 | 1077 | 1016 | 42 | 19 | ND | ND | |
| Spring | e1^a^ | 7/5 | 122.02 | 28.97 | 15.2 | 3 | 18.39 | 28.41 | 2.59 | 25 | 241 | 217 | 19 | 5.6 | ND | ND | |
| Spring | e2^a^ | 7/5 | 122.13 | 28.85 | 22.2 | 3 | 18.78 | 29.13 | 3.22 | 25 | 947 | 859 | 50 | 38 | 0.01 | 12.83 | |
| Spring | e3^a^ | 7/5 | 122.18 | 28.72 | 28.6 | 3 | 18.83 | 29.12 | 2.92 | 25 | 1504 | 1417 | 55 | 31 | ND | ND | |
| Spring | e4^a^ | 7/5 | 122.24 | 28.59 | 42.2 | 3 | 18.57 | 30.05 | 0.56 | 25 | 763 | 737 | 20 | 6 | 0.01 | 8.04 | |
| Spring | e5^a^ | 7/5 | 122.33 | 28.45 | 58.2 | 3 | 19.75 | 31.33 | 0.58 | 25 | 1738 | 1698 | 40 | 0 | ND | ND | |
| Spring | e6^a^ | 7/5 | 122.44 | 28.34 | 67.8 | 3 | 20.92 | 33.06 | 0.72 | 25 | 335 | 322 | 13 | 0 | 0.01 | ND | |
| Spring | f1^a^ | 6/5 | 121.80 | 28.31 | 16 | 3 | 18.43 | 29.06 | 2.75 | 25 | 470 | 447 | 23 | 0 | ND | ND | |
| Spring | f2^a^ | 6/5 | 121.86 | 28.15 | 29.8 | 3 | 18.77 | 29.26 | 3.84 | 25 | 1194 | 1157 | 26 | 12 | 0.01 | 8.66 | |
| Spring | f3^a^ | 6/5 | 121.92 | 28.08 | 40.4 | 3 | 18.81 | 30.10 | 3.34 | 25 | 709 | 695 | 9.6 | 4.2 | ND | ND | |
| Spring | f4^a^ | 6/5 | 122.03 | 27.96 | 56.6 | 3 | 20.26 | 32.14 | 1.19 | 25 | 742 | 726 | 16 | 0 | 0.01 | 0.91 | |
| Spring | f5^a^ | 6/5 | 122.12 | 27.87 | 73 | 3 | 21.22 | 33.23 | 1.06 | 50 | 444 | 428 | 13 | 2.5 | 0.01 | 0.05 | |
| Spring | g1^a^ | 5/5 | 121.10 | 27.71 | 17.6 | 3 | 18.18 | 28.74 | 1.43 | 25 | 136 | 125 | 7 | 3.9 | ND | ND | |
| Spring | g2^a^ | 5/5 | 121.28 | 27.59 | 29.2 | 3 | 18.81 | 30.19 | 5.23 | 25 | 1090 | 1060 | 25 | 5.2 | 0.01 | 5.73 | |
| Spring | g3^a^ | 5/5 | 121.42 | 27.50 | 39.2 | 3 | 20.16 | 31.46 | 2.30 | 25 | 1342 | 1305 | 37 | 0 | ND | ND | |
| Spring | g4^a^ | 5/5 | 121.56 | 27.41 | 50 | 3 | 21.01 | 32.47 | 2.32 | 25 | 909 | 882 | 25 | 2.8 | 0.01 | 0.57 | |
| Spring | g5^a^ | 6/5 | 121.67 | 27.35 | 54.8 | 3 | 23.03 | 34.31 | 0.47 | 50 | 155 | 124 | 8.6 | 23 | ND | ND | |
| Spring | g6^a^ | 6/5 | 121.87 | 27.22 | 85 | 3 | 22.96 | 34.11 | 0.48 | 25 | 257 | 233 | 9.7 | 14 | ND | ND | |
| Spring | g7^a^ | 6/5 | 122.07 | 27.01 | 95.4 | 3 | 24.37 | 34.53 | 0.34 | 25 | 48 | 40 | 3.3 | 4.4 | 0.01 | 0.2 | |
| Spring | h1^a^ | 5/5 | 120.53 | 27.09 | 15.2 | 3 | 19.88 | 28.36 | 3.75 | 25 | 1527 | 1491 | 36 | 0 | ND | ND | |

**Table S1** continued

| Spring | Station | Sampling date | Longitude | Latitude | Depth | Sampling depth | Temperature | Salinity | Chlorophyll *a* | Water volume | ∑PB | Brassicasterol | Dinosterol | C_37_ alkenones | DIP | NO_x_ |
| --- | --- | --- | --- | --- | --- | --- | --- | --- | --- | --- | --- | --- | --- | --- | --- | --- |
|  | name | Day/month | (E/°) | (N/°) | (m) | (m) | (℃) |  | (μg L^−1^) | (L) | (ng L^−1^) | (ng L^−1^) | (ng L^−1^) | (ng L^−1^) | (μmol L^−1^) | (μmol L^−1^) |
| Spring | h2^a^ | 5/5 | 120.61 | 27.00 | 26.2 | 3 | 19.23 | 28.78 | 2.20 | 25 | 515 | 503 | 11 | 0 | 0.01 | 10.78 |
| Spring | h3^a^ | 5/5 | 120.75 | 26.90 | 39.6 | 3 | 19.69 | 31.54 | 3.11 | 25 | 1007 | 990 | 17 | 0 | ND | ND |
| Spring | h4^a^ | 5/5 | 120.86 | 26.83 | 47.2 | 3 | 20.37 | 31.90 | 1.54 | 25 | 499 | 468 | 31 | 0 | 0.01 | 2.3 |
| Spring | h5^a^ | 4/5 | 120.98 | 26.74 | 59 | 3 | 20.89 | 32.14 | 0.39 | 25 | 770 | 752 | 13 | 5.4 | ND | ND |
| Spring | h6^a^ | 4/5 | 121.10 | 26.66 | 67.4 | 3 | 20.29 | 32.61 | 0.50 | 25 | 193 | 193 | 0 | 0 | 0.01 | ND |
| Spring | a1-1^b^ | 11/5 | 122.12 | 32.25 | 17.4 | 3 | 15.59 | 30.95 | 1.62 | 25 | 77 | 58 | 18 | 0 | 0.82 | 14.83 |
| Spring | a1-4^b^ | 11/5 | 122.74 | 32.25 | 33 | 3 | 17.53 | 26.53 | 0.78 | 25 | 932 | 918 | 14 | 0 | 0.08 | 17.86 |
| Spring | a1-7^b^ | 11/5 | 123.49 | 32.25 | 38 | 3 | 16.48 | 29.63 | 1.62 | 25 | 475 | 452 | 23 | 0 | 0.15 | 7.22 |
| Spring | a2-1^b^ | 17/5 | 122.26 | 31.95 | 18 | 3 | 16.60 | 29.70 | 21.98 | 25 | 262 | 208 | 50 | 4.3 | ND | 46.35 |
| Spring | a2-3^b^ | 17/5 | 122.75 | 31.88 | 36.1 | 3 | 18.36 | 26.02 | 0.41 | 25 | 197 | 137 | 58 | 2.6 | ND | 27.64 |
| Spring | a2-6^b^ | 17/5 | 123.49 | 31.80 | 36.3 | 3 | 16.02 | 31.30 | 0.71 | 25 | 352 | 339 | 13 | 0 | 0.07 | 7.5 |
| Spring | a3-1^b^ | 16/5 | 122.25 | 31.66 | 16.8 | 3 | 16.41 | 29.51 | 2.22 | 25 | 186 | 165 | 21 | 0 | 0.86 | 87.64 |
| Spring | a3-4^b^ | 16/5 | 122.66 | 31.59 | 33.6 | 3 | 18.26 | 26.52 | 9.37 | 25 | 667 | 479 | 177 | 11 | 0.1 | 31.78 |
| Spring | a3-6^b^ | 16/5 | 123.00 | 31.50 | 35.5 | 3 | 19.09 | 29.53 | 0.69 | 25 | 587 | 541 | 47 | 0 | 0.07 | 12.25 |
| Spring | a3-8^b^ | 16/5 | 123.49 | 31.39 | 41.2 | 3 | 18.97 | 29.17 | 2.00 | 25 | 808 | 786 | 21 | 0 | 0.06 | 15.15 |
| Spring | a3-10^b^ | 16/5 | 123.99 | 31.28 | 57.8 | 3 | 18.29 | 29.37 | 1.27 | 25 | 217 | 197 | 19 | 0 | 0.06 | 13.68 |
| Spring | a5-3^b^ | 14/5 | 122.66 | 31.06 | 22 | 3 | 18.17 | 24.07 | 1.83 | 25 | 166 | 136 | 23 | 6.8 | 1.08 | 55.28 |
| Spring | a5-7^b^ | 15/5 | 123.49 | 30.78 | 55.7 | 3 | 19.48 | 29.38 | 0.54 | 25 | 249 | 217 | 32 | 0 | 0.09 | 11.97 |
| Spring | a6-11^b^ | 13/5 | 123.99 | 30.40 | 48.7 | 3 | 19.00 | 29.99 | 2.47 | 25 | 1136 | 1082 | 54 | 0 | 0.07 | 5.33 |

**Table S1** continued

| Season | Station | Sampling date | Longitude | Latitude | Depth | Sampling depth | Temperature | Salinity | Chlorophyll *a* | Water volume | ∑PB | Brassicasterol | Dinosterol | C_37_ alkenones | DIP | NO_x_ |
| --- | --- | --- | --- | --- | --- | --- | --- | --- | --- | --- | --- | --- | --- | --- | --- | --- |
|  | name | Day/month | (E/°) | (N/°) | (m) | (m) | (℃) |  | (μg L^−1^) | (L) | (ng L^−1^) | (ng L^−1^) | (ng L^−1^) | (ng L^−1^) | (μmol L^−1^) | (μmol L^−1^) |
| Summer | a1-3^c^ | 5/8 | 122.49 | 32.26 | 27.4 | 3 | 27.99 | 28.26 | 5.81 | 25 | 106 | 83 | 8.4 | 14 | 0.11 | 14.4 |
| Summer | a2-2^c^ | 6/8 | 122.51 | 31.91 | 25.2 | 3 | 28.25 | 21.49 | 8.67 | 25 | 162 | 153 | 9.3 | 0 | 0.31 | 44.13 |
| Summer | a2-3^c^ | 6/8 | 122.76 | 31.88 | 35.6 | 3 | 27.30 | 29.71 | 0.19 | 25 | 166 | 142 | 24 | 0 | 0.4 | 47.98 |
| Summer | a2-4^c^ | 6/8 | 123.11 | 31.88 | 37.6 | 3 | 27.70 | 31.44 | 4.74 | 25 | 802 | 552 | 90 | 160 | 0.06 | 14.56 |
| Summer | a2-5^c^ | 6/8 | 123.26 | 31.90 | 41.3 | 3 | 27.94 | 31.30 | 0.54 | 25 | 1917 | 1792 | 119 | 5.8 | 0.1 | 0.39 |
| Summer | a2-6^c^ | 6/8 | 123.51 | 31.71 | 41.5 | 3 | 28.30 | 33.13 | 0.29 | 25 | 1279 | 1236 | 43 | 0 | 0.1 | 0.28 |
| Summer | a3-8^c^ | 7/8 | 123.51 | 31.39 | 40 | 3 | 28.09 | 33.60 | 1.09 | 25 | 1327 | 1236 | 49 | 42 | 0.13 | 0.65 |
| Summer | a4-5^c^ | 6/8 | 123.03 | 31.17 | 60.7 | 3 | 27.44 | 30.43 | 24.23 | 25 | 954 | 855 | 99 | 0 | 0.05 | 32.9 |
| Summer | a5-4^c^ | 7/8 | 122.81 | 31.01 | 30.3 | 3 | 26.68 | 27.20 | 2.45 | 25 | 98 | 85 | 13 | 0 | 0.68 | 36.83 |
| Summer | a5-5^c^ | 19/8 | 123.14 | 30.94 | 50 | 3 | 25.77 | 31.78 | 1.18 | 25 | 159 | 122 | 37 | 0 | 0.87 | 8.61 |
| Summer | a6-3^c^ | 7/8 | 122.38 | 30.91 | 13.7 | 3 | 25.71 | 26.91 | 0.65 | 25 | 69 | 56 | 13 | 0 | 1.37 | 74.64 |
| Summer | a6-6^c^ | 18/8 | 122.84 | 30.79 | 27 | 3 | 24.64 | 30.76 | 0.98 | 25 | 206 | 178 | 28 | 0 | 0.31 | 5.24 |
| Summer | a6-7^c^ | 18/8 | 122.99 | 30.73 | 47 | 3 | 26.69 | 32.22 | 2.81 | 25 | 173 | 138 | 34 | 0 | 0.18 | 2.47 |
| Summer | a1^c^ | 7/8 | 122.50 | 30.56 | 27.6 | 3 | 26.11 | 28.08 | 1.43 | 25 | 1525 | 1483 | 23 | 20 | 0.55 | 21.63 |
| Summer | a2^c^ | 7/8 | 122.75 | 30.49 | 40.4 | 3 | 27.24 | 25.74 | 5.31 | 25 | 858 | 746 | 13 | 99 | 0.49 | 31.64 |
| Summer | a3^c^ | 7/8 | 122.99 | 30.42 | 55 | 3 | 28.50 | 29.39 | 18.99 | 25 | 753 | 612 | 141 | 0 | 0.11 | 15.19 |
| Summer | a4^c^ | 7/8 | 123.25 | 30.35 | 57.7 | 3 | 29.44 | 32.96 | 1.03 | 25 | 790 | 757 | 32 | 0 | 0.07 | 0.84 |
| Summer | a5^c^ | 7/8 | 123.51 | 30.28 | 66.1 | 3 | 29.98 | 32.88 | 0.54 | 25 | 225 | 207 | 18 | 0 | 0.05 | 0.36 |
| Summer | b1^c^ | 8/8 | 122.36 | 30.07 | 15.4 | 3 | 27.97 | 27.32 | 3.04 | 25 | 141 | 86 | 54 | 0 | 0.77 | 23.12 |
| Summer | b2^c^ | 8/8 | 122.51 | 30.01 | 20 | 3 | 26.62 | 28.54 | 1.81 | 25 | 54 | 47 | 6.9 | 0 | 0.63 | 21.4 |
| Summer | b3^c^ | 8/8 | 122.76 | 29.93 | 42.1 | 3 | 28.74 | 30.56 | 2.75 | 25 | 1772 | 1700 | 73 | 0 | 0.1 | 3.79 |
| Summer | b4^c^ | 8/8 | 123.00 | 29.86 | 57.3 | 3 | 29.53 | 31.49 | 0.57 | 25 | 930 | 906 | 24 | 0 | 0.1 | 0.86 |
| Summer | b5^c^ | 8/8 | 123.24 | 29.78 | 61.8 | 3 | 29.72 | 32.28 | 0.20 | 25 | 435 | 407 | 24 | 4.3 | 0.06 | 0.26 |

**Table S1** continued

| Season | Station | Sampling date | Longitude | Latitude | Depth | Sampling depth | Temperature | Salinity | Chlorophyll *a* | Water volume | ∑PB | Brassicasterol | Dinosterol | C_37_ alkenones | DIP | NO_x_ |
| --- | --- | --- | --- | --- | --- | --- | --- | --- | --- | --- | --- | --- | --- | --- | --- | --- |
|  | name | Day/month | (E/°) | (N/°) | (m) | (m) | (℃) |  | (μg L^−1^) | (L) | (ng L^−1^) | (ng L^−1^) | (ng L^−1^) | (ng L^−1^) | (μmol L^−1^) | (μmol L^−1^) |
| Summer | c1^c^ | 9/8 | 122.25 | 29.62 | 14.1 | 3 | 27.02 | 29.42 | 1.99 | 25 | 97 | 55 | 42 | 0 | 0.48 | 15.93 |
| Summer | c2^c^ | 9/8 | 122.32 | 29.57 | 18.3 | 3 | 27.25 | 29.37 | 1.29 | 25 | 57 | 48 | 8.4 | 0 | 0.49 | 16.85 |
| Summer | c3^c^ | 9/8 | 122.51 | 29.50 | 31.1 | 3 | 29.17 | 30.35 | 6.43 | 25 | 639 | 221 | 383 | 34 | 0.08 | 4.68 |
| Summer | c4^c^ | 9/8 | 122.62 | 29.46 | 43.5 | 3 | 29.63 | 31.02 | 1.06 | 25 | 397 | 73 | 9.1 | 315 | 0.07 | 1.43 |
| Summer | c5^c^ | 9/8 | 122.75 | 29.42 | 51.7 | 3 | 29.47 | 30.93 | 0.67 | 25 | 389 | 220 | 81 | 88 | 0.19 | 0.58 |
| Summer | c6^c^ | 9/8 | 122.98 | 29.35 | 58.6 | 3 | 30.98 | 32.74 | 0.21 | 25 | 148 | 114 | 25 | 9.5 | 0.18 | 0.13 |
| Summer | d1^c^ | 10/8 | 122.08 | 29.22 | 11.6 | 3 | 29.62 | 30.93 | 2.61 | 25 | 118 | 50 | 68 | 0 | 0.23 | 7.56 |
| Summer | d2^c^ | 10/8 | 122.27 | 29.13 | 22 | 3 | 30.11 | 30.65 | 0.81 | 25 | 210 | 107 | 55 | 48 | 0.06 | 2.14 |
| Summer | d3^c^ | 10/8 | 122.36 | 29.10 | 31 | 3 | 30.04 | 30.99 | 2.39 | 25 | 385 | 252 | 100 | 32 | 0.07 | 1.09 |
| Summer | d4^c^ | 10/8 | 122.50 | 29.06 | 47 | 3 | 29.82 | 31.29 | 1.86 | 25 | 424 | 324 | 100 | 0 | 0.08 | 1.34 |
| Summer | d5^c^ | 9/8 | 122.63 | 29.02 | 56 | 3 | 28.22 | 30.48 | 4.04 | 25 | 383 | 285 | 93 | 4.6 | 0.07 | 0.5 |
| Summer | d6^c^ | 9/8 | 122.76 | 28.98 | 61.7 | 3 | 30.24 | 31.07 | 0.52 | 25 | 77 | 52 | 25 | 0 | 0.08 | 0.91 |
| Summer | d7^c^ | 9/8 | 122.99 | 28.87 | 70 | 3 | ND | ND | ND | 25 | 136 | 109 | 18 | 9.6 | 0.07 | 0.65 |
| Summer | e1^c^ | 10/8 | 122.02 | 28.98 | 15 | 3 | 29.30 | 31.03 | 3.44 | 25 | 157 | 55 | 102 | 0 | 0.18 | 5.03 |
| Summer | e2^c^ | 10/8 | 122.13 | 28.85 | 22.5 | 3 | 30.96 | 32.53 | 0.50 | 25 | 67 | 44 | 23 | 0 | 0.06 | 0.28 |
| Summer | e3^c^ | 10/8 | 122.18 | 28.72 | 28.2 | 3 | 30.57 | 32.01 | 0.05 | 25 | 135 | 103 | 32 | 0 | 0.08 | 0.45 |
| Summer | e4^c^ | 10/8 | 122.24 | 28.58 | 40.7 | 3 | 30.96 | 32.57 | 0.17 | 25 | 163 | 123 | 39 | 0 | 0.1 | 0.1 |
| Summer | e5^c^ | 10/8 | 122.32 | 28.45 | 53.8 | 3 | 30.58 | 33.29 | 0.15 | 25 | 146 | 114 | 32 | 0 | 0.15 | 0.28 |
| Summer | e6^c^ | 10/8 | 122.44 | 28.34 | 66.2 | 3 | 30.62 | 33.84 | 0.19 | 25 | 128 | 93 | 29 | 6.9 | 0.09 | 0.83 |
| Summer | f1^c^ | 11/8 | 121.80 | 28.31 | 17.5 | 3 | 30.47 | 32.02 | 1.39 | 25 | 278 | 187 | 89 | 2.1 | 0.08 | 0.33 |
| Summer | f2^c^ | 11/8 | 121.87 | 28.16 | 26.4 | 3 | 30.54 | 32.70 | 0.31 | 25 | 128 | 103 | 25 | 0 | 0.09 | 0.1 |
| Summer | f3^c^ | 11/8 | 121.92 | 28.08 | 38 | 3 | 30.42 | 33.86 | 0.36 | 25 | 89 | 72 | 17 | 0 | 0.09 | 0.24 |
| Summer | f4^c^ | 10/8 | 122.03 | 27.96 | 57.5 | 3 | 30.37 | 33.85 | 0.14 | 25 | 55 | 42 | 13 | 0 | 0.11 | 0.23 |

**Table S1** continued

| Season | Station | Sampling date | Longitude | Latitude | Depth | Sampling depth | Temperature | Salinity | Chlorophyll *a* | Water volume | ∑PB | Brassicasterol | Dinosterol | C_37_ alkenones | DIP | NO_x_ |
| --- | --- | --- | --- | --- | --- | --- | --- | --- | --- | --- | --- | --- | --- | --- | --- | --- |
|  | name | Day/month | (E/°) | (N/°) | (m) | (m) | (℃) |  | (μg L^−1^) | (L) | (ng L^−1^) | (ng L^−1^) | (ng L^−1^) | (ng L^−1^) | (μmol L^−1^) | (μmol L^−1^) |
| Summer | f5^c^ | 10/8 | 122.13 | 27.87 | 76 | 3 | 30.70 | 33.73 | 0.17 | 25 | 74 | 61 | 14 | 0 | 0.07 | 0.58 |
| Summer | g1^c^ | 11/8 | 121.10 | 27.71 | 13.8 | 3 | 30.11 | 32.86 | 1.72 | 25 | 676 | 504 | 172 | 0 | 0.15 | 0.26 |
| Summer | g2^c^ | 11/8 | 121.29 | 27.59 | 27.2 | 3 | 28.95 | 33.67 | 0.77 | 25 | 152 | 93 | 58 | 0 | 0.22 | 0.58 |
| Summer | g3^c^ | 13/8 | 121.42 | 27.50 | 37 | 3 | 29.22 | 33.38 | 1.82 | 25 | 204 | 126 | 78 | 0 | 0.15 | 0.47 |
| Summer | g4^c^ | 13/8 | 121.56 | 27.41 | 49 | 3 | 29.06 | 33.86 | 0.24 | 25 | 104 | 80 | 24 | 0 | 0.11 | 0.21 |
| Summer | g5^c^ | 13/8 | 121.66 | 27.36 | 56.7 | 3 | 28.63 | 33.85 | 0.46 | 25 | 80 | 60 | 20 | 0 | 0.1 | 0.17 |
| Summer | h1^c^ | 14/8 | 120.53 | 27.08 | 18.7 | 3 | 30.08 | 33.55 | 1.22 | 25 | 224 | 137 | 87 | 0 | 0.31 | 0.4 |
| Summer | h2^c^ | 14/8 | 120.61 | 27.01 | 26.2 | 3 | 29.24 | 34.02 | 0.31 | 25 | 125 | 84 | 41 | 0 | 0.19 | 0.21 |
| Summer | h3^c^ | 14/8 | 120.73 | 26.91 | 37 | 3 | 28.85 | 33.56 | 0.32 | 25 | 135 | 107 | 28 | 0 | 0.1 | 0.2 |
| Summer | h4^c^ | 14/8 | 120.86 | 26.83 | 46 | 3 | 29.43 | 33.28 | 0.19 | 25 | 142 | 121 | 21 | 0 | 0.09 | 0.38 |
| Summer | h5^c^ | 14/8 | 120.99 | 26.74 | 59.5 | 3 | 29.76 | 33.30 | 0.19 | 25 | 71 | 63 | 7.6 | 0 | 0.08 | 0.1 |

ND: No Data.

^a^ Spring Cruise MZ17SP, ^b^ Spring Cruise NORC2017-03, ^c^ Summer Cruise MZ18SU.

**Table S2** Typical dinoflagellates, diatoms and haptophytes in the coastal ECS and their capability to produce lipid biomarkers.

| **Phytoplankton species** | **Lipid biomarker** |
| --- | --- |
| Dinoflagellates | Dinosterol production |
| *Prorocentrum donghaiense*^a^ | Yes^g^ |
| *Scrippsiella trochoidea*^a^ | Yes^h^ |
| *Gymnodinium lohmann*^b^ | Yes^j^ |
| *Prorocentrum minimum*^b^ | Yes^g^ |
| *Karenia mikimotoi*^c^ | Yes^g^ |
| Diatoms | Brassicasterol production |
| *Cylindrotheca closterium*^d^ | Yes^h^ |
| *Phaeodactylum tricornutum*^e^ | Yes^g^ |
| *Skeletonema costatum*^a^ | No^h^ |
| *Pseudo-nitzschia delicatissima*^a^ | No^h^ |
| Haptophytes | C_37_ alkenones production |
| *Emiliania huxleyi*^f^ | Yes^g^ |
| *Gephyrocapsa oceanica*^f^ | Yes^k^ |

^a^(Tian et al., 2010), ^b^(Guo et al., 2011), ^c^(Zhao et al., 2013), ^d^(Zhao et al., 2019), ^e^(Chiang et al., 1999), ^f^(Luan et al., 2016), ^g^(Ding et al., 2019), ^j^(Leblond and Chapman, 2002), ^k^(Volkman et al., 1995).

Table S3 Summary of the end-member properties used in the three end-member mixing model in the bottom layer. CDW: Changjiang Diluted Water; TSW: Taiwan Strait Water; KSSW: Kuroshio Subsurface Water.

| Season | Water mass | Representative station | Layer | Longitude | Latitude | Depth | Salinity ^a^ | Temperature | NO_x_ | DIP |
| --- | --- | --- | --- | --- | --- | --- | --- | --- | --- | --- |
|  |  |  |  | (°E) | (°N) | (m) ^a^ |  | (℃) ^a^ | (μmol L^−1^) | (μmol L^−1^) |
|  | CDW ^b^ | Station b1 | Bottom | 122.36 | 30.07 | 12±1 | 27.05±0.02 | 17.62±0.001 | 33.97 | 0.21 |
| Spring | TSW ^c^ | Station TWS3 | Bottom | 119.67 | 25.00 | 40±1 | 34.24±0.02 | 22.43±0.06 | 0.02 | 0.01 |
|  | KSSW ^d^ | Station 376 | Subsurface | 126.33 | 26.74 | 200 | 34.7±0.1 | 18.9±0.1 | 3.69 | 0.55 |
|  | CDW ^b^ | Station b1 | Bottom | 122.36 | 30.07 | 11±1 | 27.66±0.06 | 27.30±0.06 | 30.68 | 1.02 |
| Summer | TSW ^e^ | Station Y33 | Bottom | 119.80 | 24.84 | 61±1 | 34.14±0.0004 | 25.46±0.0005 | 1.85 | 0.08 |
|  | KSSW ^d^ | Station 376 | Subsurface | 126.33 | 26.74 | 200 | 34.7±0.1 | 18.9±0.1 | 3.69 | 0.55 |

Water masses proportion in the bottom layer were calculated using the properties of end-member in the bottom layer.

^a^ For depth, salinity and temperature, mean values (at depth of 1−3 m above bottom for salinity and temperature) ± SD are shown. ^b^ The end member values for CDW were obtained from the present study. ^c^ The end member values for TSW in spring were obtained from cruise MZ17SP. ^d^ The end member values for KSSW were obtained from Wang et al. (2016). ^e^ The end member values for TSW in summer were obtained from cruise NORC2018−04.

**Table S4** Spearman’s correlation coefficients between nutrients (the measured values and calculated values based on water mass proportions) and chlorophyll *a* (Chl *a*) in spring of 2017 and summer of 2018.

|  | Measured NO_x_ | Measured DIP |
| --- | --- | --- |
| Spring-Chl *a* | 0.199 | 0.022 |
| Summer-Chl *a* | 0.681** | 0.199 |
|  | Calculated NO_x_ | Calculated DIP |
| Spring-Chl *a* | 0.309** | 0.348** |
| Summer-Chl *a* | 0.587** | 0.576** |

**Significant correlation at *p* < 0.01.

**Table S5** Shapiro-Wilk test of lipid biomarker concentrations (and their ratios) in the Changjiang Diluted Water-dominated region (CDWR), the Changjiang Diluted Water-Kuroshio Subsurface Water Mixing Region (C-KMR) and the Kuroshio Subsurface Water-dominated region (KSSWR).

|  | Region | ∑PB | B/∑PB | D/∑PB | A/∑PB |
| --- | --- | --- | --- | --- | --- |
| *p* | CDWR | 0.002 | 0.029 | 0.036 | 0.000 |
|  | C-KMR | 0.000 | 0.011 | 0.003 | 0.000 |
|  | KSSWR | 0.000 | 0.000 | 0.000 | 0.000 |

*p* < 0.05: abnormal distribution.

**Table S6** Levene’s test and Kruskal-Wallis rank sum test of lipid biomarkers (and their ratios) in the Changjiang Diluted Water-dominated region (CDWR), the Changjiang Diluted Water-Kuroshio Subsurface Water Mixing Region (C-KMR) and the Kuroshio Subsurface Water-dominated region (KSSWR).

|  |  | ∑PB | B/∑PB | D/∑PB | A/∑PB |
| --- | --- | --- | --- | --- | --- |
| Levene’s test | *p^a^* | 0.016 | 0.001 | 0.000 | 0.945 |
| Kruskal-Wallis rank sum test | *p^b^* | 0.072 | 0.019 | 0.021 | 0.097 |

^a^ *p* < 0.05 indicates that data in three subregions did not conform to homogeneity of variance.

^b^ *p* < 0.05 indicates significant differences between the three subregions.

**Table** **S7** Mean values (ranges) of hydrographic parameters, nutrients, and lipid biomarkers and Chlorophyll *a* (μg L^–1^) in spring 2017 and summer 2018. ∑PB (ng L^−1^): brassicasterol + dinosterol + C_37_ alkenones; B (ng L^−1^): brassicasterol; D (ng L^−1^): dinosterol; A (ng L^−1^): C_37_ alkenones; NO_x_ (μmol L^−1^): NO_3_^−^+ NO_2_^−^; Si (μmol L^−1^): silicate; DIP (μmol L^−1^): phosphate; N/P: NO_x_ /DIP.

|  | ∑PB | Chl *a* | B | D | A | B/∑PB | D/∑PB | A/∑PB | DIP | NO_x_ | P/N |
| --- | --- | --- | --- | --- | --- | --- | --- | --- | --- | --- | --- |
|  | (ng L^−1^) | (μg L^–1^) | (ng L^−1^) | (ng L^−1^) | (ng L^−1^) |  |  |  |  |  |  |
| Spring | 863 | 2.29 | 805 | 50 | 8.0 | 0.879 | 0.108 | 0.013 | 0.16 | 17.79 | 0.014 |
|  | (20−8589) | (0.23−21.98) | (6.7−8184) | (0−346) | (0−59) | (0.343−1) | (0−0.657) | (0−0.146) | (0.01−1.08) | (0.05−87.64) | (0.0008−0.2) |
| Summer | 381 | 2.27 | 313 | 51 | 16 | 0.756 | 0.207 | 0.037 | 0.22 | 8.20 | 0.24 |
|  | (54−1917) | (0.05−24.23) | (42−1792) | (6.9−383) | (0−315) | (0.183−0.974) | (0.015−0.648) | (0−0.794) | (0.05−1.37) | (0.10−74.64) | (0.008−1.34) |

**Table S8** The mean values of water mass proportions, lipid biomarker concentrations and their ratios in the Changjiang Diluted Water-dominanted region (CDWR), the Changjiang Diluted Water-Kuroshio Subsurface Water Mixing Region (C-KMR) and the Kuroshio Subsurface Water-dominated region (KSSWR). Outliers of ∑PB were excluded (1772 and 1527 ng/L in the CDWR, 8589 ng/L in the C-KMR and 3493 ng/L in the KSSWR). ∑PB: brassicasterol + dinosterol + C_37_ alkenones; B: brassicasterol; D: dinosterol; A: C_37_ alkenones.

|  | **CDW**  **(%)** | **TWCW**  **(%)** | **KSSW**  **(%)** | **∑PB**  **(ng L^-1^)** | **B/∑PB**  **(%)** | **D/∑PB**  **(%)** | **A/∑PB**  **(%)** |
| --- | --- | --- | --- | --- | --- | --- | --- |
| **CDWR** | 83.3 | 16.0 | 0.7 | 370 | 76.9 | 22.0 | 1.2 |
| **C-KMR** | 69.0 | 20.4 | 10.6 | 949 | 89.6 | 8.7 | 1.7 |
| **KSSWR** | 51.0 | 24.9 | 24.1 | 704 | 89.8 | 8.9 | 1.3 |

**References**

Cao Y, Bi R, Wang X, Chen X, Hu J, Wang Y, Jiang, Y, Zhang H, Xing L, Zhao M (2022) The sources and burial of marine organic carbon in the eastern China marginal seas. Frontiers in Marine Science 9:1–19. https://doi.org/10.3389/fmars.2022.824181

Chiang KP, Chen YT, Gong GC (1999) Spring distribution of diatom assemblages in the East China Sea. Marine Ecology Progress Series 186:75–86. https://doi.org/10.3354/meps186075

Ding Y, Bi R, Sachs JP, Chen X, Zhang H, Li L, Zhao M (2019) Lipid biomarker production by marine phytoplankton under different nutrient and temperature regimes. Organic Geochemistry 131:34–49. https://doi.org/10.1016/j.orggeochem.2019.01.008

Geng H, Yu R, Chen Z, Peng Q, Yan T, Zhou M-J (2017) Analysis of sterols in selected bloom-forming algae in China. Harmful Algae 66:29–39. https://doi.org/10.1016/j.hal.2017.04.008

Guo S, Sun J, Zhao Q, Feng Y, Huang D, Liu S (2016) Sinking rates of phytoplankton in the Changjiang (Yangtze River) Estuary: A comparative study between *Prorocentrum dentatum* and *Skeletonema dorhnii* bloom. Journal of Marine Systems 154:5–14. https://doi.org/10.1016/j.jmarsys.2015.07.003

Guo S, Tian W, Dai M, Liu Z, Sun J (2011) Phytoplankton assemblages in the East China Sea in summer 2009. Advances in Marine Science 29:474-486. https://doi.org/10.3969/j.issn.1671-6647.2011.04.007

Jiang Z, Chen J, Zhou F, Shou L, Chen Q, Tao B, Yan X, Wang K (2015) Controlling factors of summer phytoplankton community in the Changjiang (Yangtze River) Estuary and adjacent East China Sea shelf. Continental Shelf Research 101:71–84. https://doi.org/10.1016/j.csr.2015.04.009

Leblond JD, Chapman PJ (2002) A survey of the sterol composition of the marine dinoflagellates Karenia Brevis, Karenia mikimotoi, and Karlodinium micrum: distribution of sterols within other members of the class dinophyceae. Journal of Phycology 38:670–682. https://doi.org/10.1046/j.1529-8817.2002.01181.x

Luan Q, Liu S, Zhou F, Wang J (2016) Living coccolithophore assemblages in the Yellow and East China Seas in response to physical processes during fall 2013. Marine Micropaleontology 123:29–40. https://doi.org/10.1016/j.marmicro.2015.12.004

Tian W, Sun J, Fan X, Wang M (2010) Phytoplankton Community in Coastal Waters of the East China Sea in sping 2008. Advances in Marine Science 28:170–178. https://doi.org/10.1631/jzus.A1000244

Volkman JK, Barrerr SM, Blackburn SI, Sikes EL (1995) Alkenones in Gephyrocapsa oceanica: Implications for studies of paleoclimate. Geochimica et Cosmochimica Acta 59:513–520. https://doi.org/10.1016/0016-7037(95)00325-T

Zhao R, Sun J, Song S (2013) Phytoplankton in the Yangtze River Estuary and its adjacent waters in spring 2006. Marine Science Bulletin 32:421-428. https://doi.org/10. 11840/j. issn. 1001-6392. 2013. 04. 009

Zhao Y, Yu RC, Kong FZ, Zhang QC, Geng HX, Dai L, Wang J, Zhou M (2019) Features of Phytoplankton Communities and Their Controlling Factors in the Yellow Sea and the East China Sea in Summer Time. Oceanologia et Limnologia Sinica 50:838–850. https://doi.org/10.11693/hyhz20181100268
